# First‐Line Third‐Generation EGFR Tyrosine Kinase Inhibitor Monotherapy for Advanced EGFR‐Mutated Non‐Small Cell Lung Cancer: A Systematic Review and Network Meta‐Analysis

**DOI:** 10.1002/mco2.70393

**Published:** 2025-11-06

**Authors:** Wei Du, Anlin Li, Bijing Xiao, Yunpeng Yang, Wenfeng Fang, Yan Huang, Shaodong Hong, Li Zhang

**Affiliations:** ^1^ State Key Laboratory of Oncology in South China Guangdong Provincial Clinical Research Center For Cancer Sun Yat‐sen University Cancer Center Guangzhou China; ^2^ Department of Medical Oncology Sun Yat‐sen University Cancer Center Guangzhou China

**Keywords:** Bayesian network meta‐analysis, epidermal growth factor receptor, first‐line, non‐small cell lung cancer, tyrosine kinase inhibitors

## Abstract

Third‐generation (third‐gen) epidermal growth factor receptor (EGFR) tyrosine kinase inhibitors (TKIs) have revolutionized the management of advanced EGFR‐mutated non‐small cell lung cancer (NSCLC). However, a head‐to‐head comparison of efficacy and safety among third‐gen EGFR TKIs is lacking. Seven randomized controlled trials with 3012 patients were included. All third‐gen TKIs significantly prolonged progression‐free survival (PFS) compared to first‐generation (first‐gen) TKIs, with no significant differences in PFS or objective response rate among the third‐gen TKIs. Furmonertinib ranked highest for PFS (HR, 0.82; 95% credible intervals [CrI], 0.72–0.94). Aumolertinib demonstrated the best intracranial control (HR, 0.74; 95% CrI, 0.63–0.89). Osimertinib (HR, 0.90; 95% CrI, 0.83–0.99) and lazertinib (HR, 0.89; 95% CrI, 0.79–1.00) showed overall survival benefits over first‐gen TKIs. Furmonertinib, aumolertinib, and osimertinib had lower rates of severe treatment‐related adverse events (TRAEs), while befotertinib exhibited the highest risk of grade ≥3 TRAEs (RR, 3.96; 95% CrI, 2.35–7.17). This study is the first head‐to‐head comparison of third‐gen EGFR‐TKIs using a Bayesian network meta‐analysis, offering critical insights into efficacy and safety. Our results support personalized selection of third‐gen EGFR TKIs for patients with advanced EGFR‐mutated NSCLC, particularly for subpopulations with CNS metastases or different mutation subtypes.

## Graphical abstract

Third‐generation EGFR‐TKIs are the current first‐line standard for advanced EGFR‐mutated NSCLC. While all improve PFS versus first‐generation TKIs, no significant PFS or ORR differences exist among them. Furmonertinib, aumolertinib, and osimertinib show better tolerability, with distinct toxicity profiles. These findings may guide decision‐making between clinicians and patients in selecting the most appropriate therapeutic option.

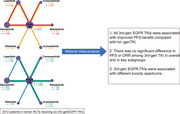

## 1 Introduction

Lung cancer is the leading cause of cancer‐related death worldwide with non‐small cell lung cancer (NSCLC) accounting for approximately 85% of all cases [1]. Among NSCLC patients, epidermal growth factor receptor (EGFR) mutations, particularly exon 19 deletions and L858R mutation, are among the most critical targets [2, 3]. These mutations are present in approximately 40%–50% of east Asian and 15%–20% in Western NSCLC patients [4]. Besides, brain metastases are common in EGFR‐mutated NSCLC patients, with 25% of newly diagnosed patients having brain metastases at diagnosis, with nearly half developing them during the disease course [5, 6].

While first‐ and second‐generation (first‐ and second‐gen) EGFR tyrosine kinase inhibitors (TKIs) have been effective in treating NSCLC, their efficacy is hampered by the emergence of resistance mutations, particularly the EGFR T790M mutation, as well as suboptimal central nervous system (CNS) penetration [7, 8]. To address these limitations, third‐generation (third‐gen) EGFR‐TKIs were developed, offering broader spectrum of efficacy, greater blood‐brain barrier penetration, and higher specificity for EGFR‐activating and resistance mutations. These agents, including osimertinib, aumolertinib, furmonertinib, befotertinib, lazertinib, and rilertinib, have since become the new standard first‐line treatment for patients with advanced EGFR‐mutated NSCLC [9, 10, 11, 12, 13, 14, 15, 16, 17].

Despite the established clinical utility of third‐gen EGFR TKIs, selecting the optimal first‐line agent remains a significant challenge for clinicians. To date, only one randomized controlled trial (RCT) has directly compared third‐gen TKIs head‐to‐head. Most existing evidence derives from individual RCTs comparing third‐gen TKIs with first‐gen agents (e.g., gefitinib or erlotinib) [18].

Additionally, while these drugs share a common mechanism of action, they exhibit distinct pharmacokinetic properties, toxicity profiles, and efficacy across subpopulations. Critical knowledge gaps persist, particularly in clinically relevant subgroups such as patients with CNS metastases or different EGFR mutation subtypes (e.g., exon 19 deletions vs. L858R). Current RCTs and indirect comparisons often lack sufficient subgroup‐specific data, further complicating personalized treatment decisions. Consequently, a comprehensive, evidence‐based evaluation of third‐gen EGFR TKIs is urgently needed. Such an analysis should integrate both direct and indirect evidence while accounting for subgroup variability to inform optimal therapeutic strategies.

To address this critical evidence gap, we conducted a comprehensive Bayesian network meta‐analysis (NMA), which is the first study to evaluate the comparative efficacy and safety of third‐gen TKI monotherapies in advanced, treatment‐naive, EGFR‐mutated NSCLC. Unlike previous NMAs that focused on comparisons between all generations of TKIs (first‐, second‐, and third‐gen), this study exclusively focuses on the comparison within the third‐gen TKIs, a clinically relevant question that remains unresolved. By incorporating the latest published trials and the most up‐to‐date data, Bayesian NMA allows for the synthesis of both direct and indirect comparisons, providing robust and comprehensive treatment rankings that are not achievable with traditional meta‐analysis methods.

## 2 Results

### 2.1 Study Characteristics

**FIGURE 1:**
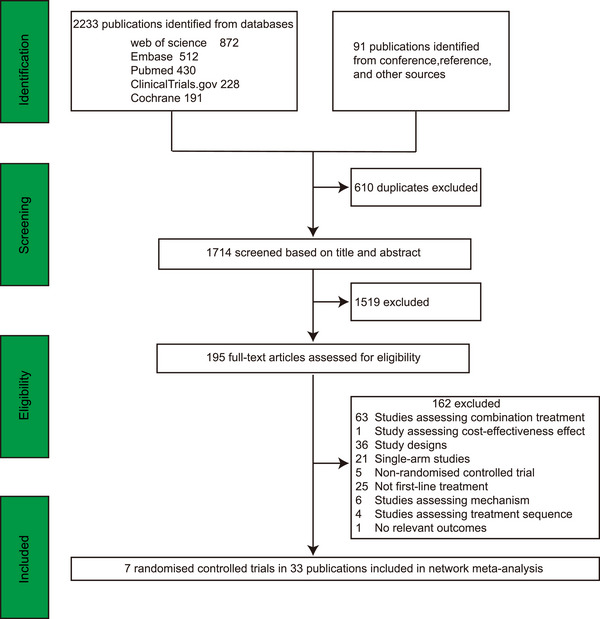
Study flow diagram. The study process followed the PRISMA guidelines.

**FIGURE 2:**
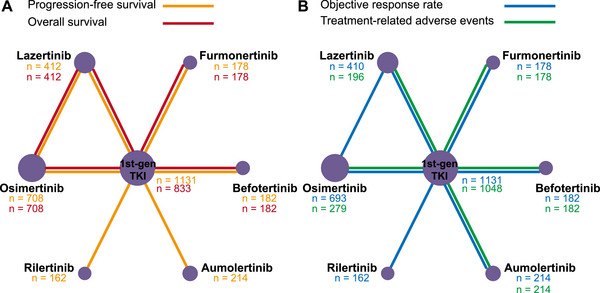
Comparative network plots for different outcomes of treatments in patients with advanced EGFR‐mutated NSCLC. Network plots illustrating the direct and indirect comparisons for (A) progression‐free survival, overall survival, and (B) objective response rate and treatment‐related adverse events. Circular nodes represent treatment strategies with the total number of involved participants below. Lines represent the direct comparisons, with thicknesses proportional to the number of involved studies^a^. ^a^All lines represent one study involved. EGFR, epidermal growth factor receptor; NSCLC, non‐small‐cell lung cancer.

Through literature search, we identified 1714 records from the initial title and abstract screening, leaving 195 studies for full‐text review. Finally, 33 eligible publications reporting seven RCTs were included in the systematic review and NMA (Figure 1). There were three RCTs conducted worldwide and four RCTs conducted in mainland China. Five trials used gefitinib as the control treatment. Only one trial used icotinib as the control group. Two trials assessed osimertinib and lazertinib. There was one study each assessing aumolertinib, furmonertinib, befotertinib, and rilertinib. We assumed that three first‐gen EGFR TKIs showed the same outcomes when compared with third‐gen EGFR TKIs, respectively. Table 1 summarizes the main characteristics of all studies involving 3012 patients. All studies were multi‐center and published between 2018 and 2024. Most of the studies reported comparisons of efficacy and safety between third‐gen TKIs and first‐gen TKIs. Only one RCT directly compared third‐gen TKI with another third‐gen TKI. The network diagram is presented in Figure 2.

**TABLE 1:** Baseline characteristics of studies included in the network meta‐analysis.

				EGFR mutation				
Study (phase, ethnicity)	Sample size (No); median age	Female (%)	CNS metastases (%)	Exon 19 deletion%	Leu 858 Arg%	Intervention arm	Control arm	Reported outcomes
FLAURA 2018 (III, multiple)	279/277; 64/64	64/62	19/23	63/63	37/37	Osimertinib 80 mg once daily	Gefitinib 250 mg once daily	PFS, OS, ORR^a^, intracranial PFS, TRAE, grade ≥3 TRAEs
							Erlotinib 150 mg once daily	
AENEAS 2022 (III, mainland China)	214/215; 59/62	63/63	26/27	65/66	35/34	Aumolertinib 110 mg once daily	Gefitinib 250 mg once daily	PFS, ORR^a^, intracranial PFS, TRAE,
FURLONG 2022 (III, mainland China)	178/179; 59/60	65/62	35/32	51/51	49/49	Furmonertinib 80 mg once daily	Gefitinib 250 mg once daily	PFS, OS, ORR, intracranial PFS, TRAE, grade ≥3 TRAEs
LASER301 2023 (III, multiple)	196/197 67/64	67/60	26/24	62/62	38/38	Lazertinib 240 mg once daily	Gefitinib 250 mg once daily	PFS, OS, ORR, intracranial PFS, TRAE, grade ≥3 TRAEs
Lu et al. 2023 (III, mainland China)	182/180 60/58	60/60	26/25	64/65	36/35	Befotertinib 75–100 mg once daily	Icotinib 125 mg three times per day	PFS, OS, ORR, intracranial PFS, TRAE, grade ≥3 TRAEs
MARIPOSA 2024 (III, multiple)	429/216 63/63	59/63	40/40	60/61	40/39	Osimertinib 80 mg once daily	Lazertinib 240 mg once daily	PFS, OS, ORR,
SHC013‐III‐01 2024 (III, mainland China)	162/83 64/62	58/61	33/35	NA	NA	Rilertinib 200 mg once daily	Gefitinib 250 mg once daily	PFS, ORR, grade ≥3 TRAEs

^a^ Investigator assessment.

Abbreviations: CNS, central nervous system; EGFR, epidermal growth factor receptor; PFS, progression‐free survival; OS, overall survival; ORR, objective response rate; TRAE, treatment‐related adverse event.

Figure S1 summarizes the detailed risk of bias assessments. Only one RCT was at high risk of bias, due to inadequate blinding of participants and personnel arising from its open‐label study designs.

### 2.2 Network Meta‐Analysis

All third‐gen TKIs were included in the analysis of progression‐free survival (PFS) and objective response rate (ORR), four third‐gen TKIs including furmonertinib, befotertinib, osimertinib, and lazertinib were analyzed for overall survival (OS), and five third‐gen TKIs except for rilertinib for intracranial PFS. Data on treatment‐related adverse events (TRAEs) and grade ≥ 3 TRAEs were available for five third‐gen TKIs. Rilertinib lacked data on TRAEs, while aumolertinib lacked data on grade ≥ 3 TRAEs. We used updated data from trials with longer follow‐up if available.

#### 2.2.1 Efficacy

**FIGURE 3:**
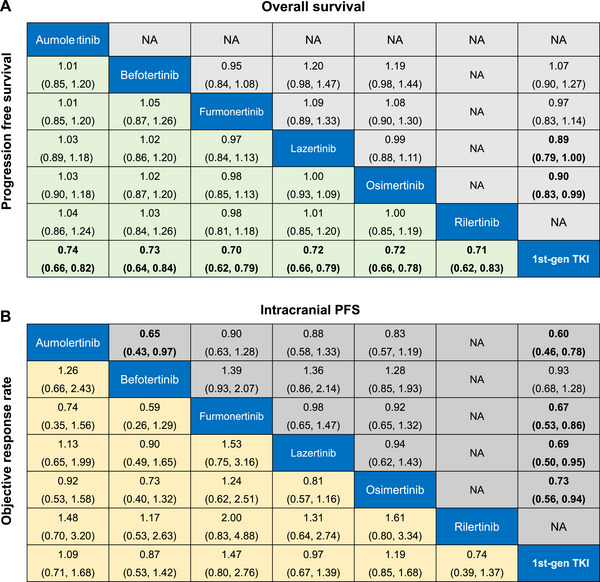
Pooled estimates of the network meta‐analysis. (A) Pooled hazard ratios (95% credible intervals) for OS (upper triangle^a^) and PFS (lower triangle^b^). (B) Pooled odds ratios (95% credible intervals) for intracranial PFS (upper triangle^a^) and ORR (lower triangle^b^). ^a^The results are presented as row‐defined treatment versus column‐defined treatment. ^b^The results are presented as column‐defined treatment versus row‐defined treatment. ORR, objective response rate; OS, overall survival; PFS, progression‐free survival.

In terms of PFS (Figure 3A), the results of NMA showed a significant advantage of third‐gen TKIs compared with first‐gen TKIs. No significant differences were found among all third‐gen TKIs. Nearly all third‐gen TKIs significantly prolonged intracranial PFS compared with first‐gen TKIs, except the befotertinib (HR, 0.93; 95% credible intervals [CrI] 0.68–1.28). Aumolertinib (HR, 0.65; 95% CrI, 0.43–0.97) showed a significant improvement in intracranial PFS compared with Befotertinib (Figure 3B).

In terms of OS (Figure 3A), osimertinib (HR, 0.90; 95% CrI 0.83 to 0.99) and lazertinib (HR, 0.89; 95% CrI 0.79–1.00) demonstrated significant improvements compared to first‐gen TKIs, though the upper bounds approach 1, indicating borderline significance. Preliminary results showed that befotertinib (HR 1.07; 95% CrI 0.90–1.27) and furmonertinib (HR, 0.97; 95% CrI 0.83 to 1.14) did not prolong the OS over the first‐gen EGFR TKIs.

In terms of ORR, no significant difference was observed between third‐gen EGFR TKIs and first‐gen EGFR TKIs or among the third‐gen EGFR‐TKI monotherapies (Figure 3B).

#### 2.2.2 Safety

**FIGURE 4:**
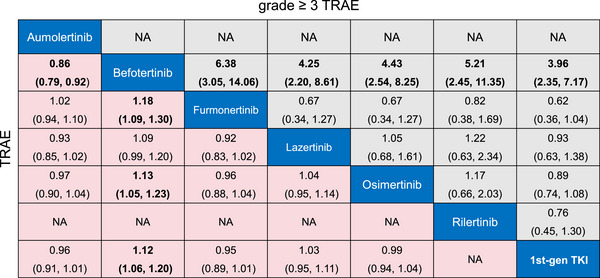
Safety profiles of third‐gen TKIs. Pooled risk ratios (95% credible intervals) for grade ≥ 3 TRAEs (upper triangle^a^) and TRAEs (lower triangle^b^). ^a^The results are presented as row‐defined treatment versus column‐defined treatment. ^b^The results are presented as column‐defined treatment versus row‐defined treatment. TKIs, tyrosine kinase inhibitors; TRAEs, treatment‐related adverse events.

Compared to first‐gen EGFR TKIs, Befotertinib was associated with a higher risk of any‐grade TRAEs (RR, 1.12; 95% CrI, 1.06–1.20) and grade ≥ 3 TRAEs (RR, 3.96; 95% CrI, 2.35–7.17). No significant differences in any‐grade TRAEs or grade ≥ 3 TRAEs were observed for osimertinib, aumolertinib, furmonertinib, rilertinib, or lazertinib compared to first‐gen EGFR TKIs. In comparisons among third‐gen TKIs, befotertinib exhibited a higher risk of any‐grade TRAEs compared to furmonertinib (RR, 1.18; 95% CrI, 1.09–1.30), osimertinib (RR, 1.13; 95% CrI, 1.05–1.23), and aumolertinib (RR, 1.17; 95% CrI, 1.08–1.27). For grade ≥ 3 TRAEs, befotertinib consistently demonstrated the highest risk compared to furmonertinib (RR, 6.38; 95% CrI, 3.05–14.06), osimertinib (RR, 4.43; 95% CrI, 2.54–8.25), lazertinib (RR, 4.25; 95% CrI, 2.20–8.61), and rilertinib (RR, 5.21; 95% CrI, 2.45–11.35) (Figure 4).

We further analyzed six commonly reported TRAEs for EGFR TKIs, including rash, diarrhea, stomatitis, elevated creatine phosphokinase (CPK), elevated alanine aminotransferase (ALT), and thrombocytopenia (Table 2). Compared to first‐gen TKIs, third‐gen TKIs were generally associated with a lower risk of rash and elevated ALT but a higher risk of elevated CPK and thrombocytopenia. Among the third‐gen TKIs, aumolertinib and furmonertinib exhibited generally lower risks of rash and/or diarrhea compared to befotertinib, osimertinib, and rilertinib. Notably, compared with aumolertinib, furmonertinib demonstrates a lower risk of CPK elevation. Osimertinib demonstrated the lowest risk for elevated ALT. Befotertinib generally had a worse safety profile regarding elevated ALT and thrombocytopenia compared to other third‐gen TKIs but appeared to have a lower risk of stomatitis. Rilertinib had a moderate overall safety profile but presented the highest risk of diarrhea among the third‐gen TKIs.

**TABLE 2:** Relative toxicity of treatments on six commonly reported specific adverse events for third‐gen EGFR‐TKIs.

	Rash	Diarrhea	Stomatitis	Elevated creatine phosphokinase	Elevated ALT	Thrombocytopenia
**Comparison**	**vs. Aumolertinib**					
Befotertinib	1.73 (1.13, 2.68)	1.48 (0.75, 2.89)	NA	0.54 (0.16, 2.19)	1.73 (1.19, 2.52)	4.41 (1.75, 11.79)
Furmonertinib	0.85 (0.52, 1.37)	1.58 (0.96, 2.64)	NA	0.14 (0.04, 0.44)	0.93 (0.65, 1.33)	0.59 (0.21, 1.67)
Osimertinib	1.51 (0.90, 2.56)	2.03 (1.34, 3.15)	NA	NA	0.53 (0.29, 0.92)	NA
Rilertinib	0.74 (0.41, 1.32)	3.70 (2.25, 6.31)	NA	0.80 (0.27, 2.91)	0.83 (0.50, 1.37)	0.52 (0.18, 1.67)
First‐gen TKI	1.97 (1.46, 2.72)	2.10 (1.43, 3.16)	NA	0.19 (0.10, 0.31)	1.98 (1.56, 2.55)	0.33 (0.17, 0.59)
**Comparison**	**vs. Befotertinib**					
Furmonertinib	0.49 (0.30, 0.79)	1.07 (0.58, 2.02)	2.93 (1.06, 8.60)	0.27 (0.05, 1.17)	0.54 (0.36, 0.78)	0.13 (0.05, 0.39)
Osimertinib	0.87 (0.52, 1.46)	1.37 (0.79, 2.44)	3.19 (1.35, 8.22)	NA	0.31 (0.17, 0.54)	NA
Rilertinib	0.43 (0.24, 0.76)	2.51 (1.35, 4.80)	1.88 (0.59, 6.53)	1.49 (0.30, 7.46)	0.48 (0.28, 0.81)	0.12 (0.04, 0.39)
First‐gen TKI	1.14 (0.85, 1.55)	1.42 (0.84, 2.47)	2.20 (0.99, 5.35)	0.35 (0.09, 1.03)	1.15 (0.87, 1.52)	0.07 (0.03, 0.14)
**Comparison**	**vs. Furmonertinib**					
Osimertinib	1.78 (1.02, 3.13)	1.28 (0.90, 1.83)	1.10 (0.53, 2.22)	NA	0.57 (0.31, 1.00)	NA
Rilertinib	0.87 (0.47, 1.61)	2.33 (1.50, 3.73)	0.64 (0.23, 1.89)	5.58 (1.43, 25.99)	0.90 (0.53, 1.49)	0.88 (0.27, 3.12)
First‐gen TKI	2.32 (1.63, 3.41)	1.33 (0.97, 1.83)	0.76 (0.40, 1.40)	1.29 (0.48, 3.58)	2.13 (1.66, 2.80)	0.55 (0.24, 1.20)
**Comparison**	**vs. Osimertinib**					
Rilertinib	0.49 (0.26, 0.93)	1.82 (1.28, 2.68)	0.58 (0.24, 1.52)	NA	2.13 (1.66, 2.80)	NA
First‐gen TKI	1.31 (0.86, 2.00)	1.04 (0.88, 1.22)	0.69 (0.49, 0.96)	NA	3.72 (2.29, 6.45)	NA
**Comparison**	**vs. Rilertinib**					
First‐gen TKI	2.66 (1.65, 4.39)	0.57 (0.40, 0.78)	1.18 (0.48, 2.71)	0.24 (0.07, 0.58)	2.38 (1.54, 3.73)	0.63 (0.23, 1.45)

*Note*: Pooled risk ratios for each available comparison on each specific adverse event (any grade). Significant values are in colored in gray (more toxicity) and light yellow (less toxicity).

Abbreviations: ALT, alanine aminotransferase; EGFR, epidermal growth factor receptor; TKIs, tyrosine kinase inhibitors.

### 2.3 Bayesian Ranking Profiles

Figure S2 shows the Bayesian ranking profiles of comparable treatments for each outcome. Of note, furmonertinib ranked first for PFS (SUCRA = 0.72), ORR (SUCRA = 0.85), and any grade TRAEs (SUCRA = 0.88). Lazertinib had the highest probability of ranking first in OS (SUCRA = 0.83). Aumolertinib ranked first for intracranial PFS. Befotertinib ranked last for any grade TRAEs.

### 2.4 Subgroup Analysis

Due to insufficient data, subgroup analyses were only conducted for PFS across five third‐gen TKIs (osimertinib, aumolertinib, furmonertinib, befotertinib, and lazertinib). We considered subgroups with data available for all five drugs (Figures S3–S7).

All included third‐gen TKIs consistently demonstrated longer PFS compared to first‐gen TKIs across various subgroups, including EGFR mutation types (19del or L858R), ECOG PS (0 or 1), sex (male or female), smoking history (never smokers or former/current smokers), and CNS metastases (with or without), except for befotertinib, which failed to show a benefit in the L858R subgroup. While no significant PFS differences were observed among the third‐gen TKIs within these subgroups, furmonertinib ranked first among patients in the 19del subgroup, ECOG 0 subgroup, male subgroup, and in patients without CNS metastases subgroup. Aumolertinib ranked first in the female subgroup, never‐smoking subgroup, and in patients with brain metastases. Lazertinib ranked first in the L858R subgroup, ECOG 1 subgroup, and former/current smoker subgroup.

### 2.5 Quality of Evidence

The fit of the consistency model was similar or better than that of inconsistency model (Table S1). The majority of direct treatment comparisons were based on evidence from a single trial; as a result, heterogeneity could not be assessed for these comparisons. Forest plots of pairwise versus network comparisons with heterogeneity estimates are shown in Figures S8–S10. We found minimal (*I* ^2^ = 0%) heterogeneity in the OS, PFS, and ORR networks. We also noted a concordance between direct and indirect evidence by comparing outcomes derived from pairwise and network meta‐analyses. The node splitting analysis revealed no significant discrepancies between direct and indirect estimates when evaluating PFS, OR, and ORR (Table S2). Figures S11–S14 show the convergence of the Markov chain Monte Carlo chains established by the history feature and the Brooks–Gelman–Rubin diagnostic for OS, PFS, ORR, and any grade TRAEs.

## 3 Discussion

The advent of multiple third‐gen EGFR TKIs has dramatically transformed the treatment landscape for NSCLC while simultaneously complicating decision‐making for both patients and clinicians [19, 20]. To our knowledge, this is the first study specifically focused on comparing the efficacy and toxicity of the third‐gen EGFR TKIs (osimertinib, aumolertinib, furmonertinib, befotertinib, lazertinib, and rilertinib) in treatment‐naive patients with advanced EGFR‐mutated NSCLC. Our findings offer the first evidence‐based, head‐to‐head comparative ranking of these agents, providing actionable guidance for clinicians in selecting optimal first‐line treatment for patients.

The landmark network meta‐analysis by Zhao et al. compared first‐line treatments for EGFR‐mutated NSCLC, including first‐/second‐gen TKIs (e.g., gefitinib and afatinib), chemotherapy combinations, and the sole third‐gen TKI available at the time (osimertinib). Their study established that osimertinib and gefitinib‐pemetrexed chemotherapy provided the best PFS benefits overall, with differential efficacy in key subgroups: Osimertinib was superior for exon 19 deletions, while gefitinib‐pemetrexed excelled in L858R mutations. However, their analysis could not address contemporary questions about comparative efficacy among third‐gen TKIs, as only osimertinib had been evaluated in randomized trials. Our study advances this evidence by focusing exclusively on six third‐gen TKIs (osimertinib, aumolertinib, furmonertinib, befotertinib, lazertinib, and rilertinib) in treatment‐naïve patients. Our findings demonstrate that furmonertinib ranks highest for PFS in patients with EGFR exon 19 deletions, whereas lazertinib shows superior efficacy in those harboring L858R mutations, further refining the precision oncology approach proposed by Zhao et al. and providing actionable data for genotype‐guided therapy. Furthermore, our analysis incorporates intracranial PFS, a critical endpoint absent in prior meta‐analyses, revealing aumolertinib's superiority in patients with brain metastases. These findings address unmet needs in modern clinical practice, where third‐gen TKIs are increasingly used as first‐line monotherapies.

Our findings show similar PFS improvement with different third‐gen TKIs in subgroups previously considered to have lower TKI efficacy, such as patients with EGFR L858R mutations or brain metastases. However, caution should be exercised, as the advantages over first‐gen TKIs do not negate the fact that these subgroups still derive less benefit from current third‐gen TKIs. For example, in the FLAURA study, the median PFS for L858R patients receiving osimertinib (14.4 months) was significantly shorter than that of the 19del subgroup (21.4 months). Patients with L858R mutations or CNS metastases might benefit from more potent combinations, such as third‐gen TKI plus chemotherapy, as evidenced by the FLAURA2 trial [21]. However, this comes at the cost of a higher risk of grade ≥ 3 TRAEs. The mechanisms underlying lower TKI sensitivity in these subgroups are only partially understood. For L858R mutations, a higher frequency of co‐mutations (e.g., TP53) might contribute [22, 23, 24]. A deeper characterization of tumor‐intrinsic features and the tumor microenvironment will further comprehensively identify potential resistance mechanisms. Subgroup findings for some monotherapy (e.g., befotertinib in L858R patients) are exploratory and require validation in larger cohorts.

In our analysis, a significant OS benefit over first‐gen TKIs was observed for osimertinib and lazertinib but not for befotertinib or furmonertinib. However, accurately assessing the true OS effects remains challenging due to several factors: (1) variability in treatment sequences across studies. For patients with unknown resistance mechanisms, subsequent‐line therapies may include chemotherapy (with or without immunotherapy/anti‐angiogenic agents), amivantamab plus chemotherapy, or antibody‐drug conjugates [25, 26, 27]; (2) limited follow‐up duration for most third‐gen TKIs; (3) potential crossover effects; and (4) the scarcity of mature OS data (currently available only for osimertinib, while aumolertinib and rilertinib lack reported outcomes) and insufficient sample sizes in some comparison groups. Understanding the resistance mechanisms of first‐line therapy is crucial for translating PFS benefits into OS improvements. Approximately 50% of patients do not exhibit clear genetic mutations at resistance [28], leaving precision strategies unavailable and suggesting a complex resistance trajectory that likely involves non‐genetic factors. Future research should focus on exploring the dynamics of transcriptomic and epigenetic clonal changes during the course of EGFR‐TKI treatment.

EGFR‐TKIs are typically not curative for the majority of patients, making quality of life a paramount consideration. We found that furmonertinib, aumolertinib, and osimertinib exhibit relatively better safety profiles. The risk of any‐grade TRAEs and grade ≥ 3 TRAEs was higher with befotertinib compared to other third‐gen TKIs. Although befotertinib demonstrates comparable efficacy to other third‐gen EGFR TKIs, its higher toxicity risk—particularly severe TRAEs leading to treatment interruptions and reduced quality of life—may limit its clinical utility, especially in patients with comorbidities or low toxicity tolerance. This safety profile should be carefully balanced against its PFS benefits when selecting first‐line therapy. Moreover, the major types of TRAEs differ among the third‐gen EGFR TKIs. The TKI structure optimization brings pros and cons. Specifically, befotertinib was associated with a higher risk of elevated ALT and thrombocytopenia. The risk of elevated CPK was higher in patients receiving aumolertinib and rilertinib. Osimertinib and furmonertinib may more frequently cause stomatitis. Rilertinib ranked highest for the risk of diarrhea. These results may help tailor treatment according to a patient's condition.

However, the interpretation of safety outcomes requires caution due to incomplete data. For example, the absence of TRAEs data for rilertinib precludes its inclusion in the comparative safety analysis, which may bias the overall safety rankings. Besides, the missing grade ≥ 3 TRAEs data for aumolertinib restrict our ability to fully evaluate its toxicity profile, particularly for severe adverse events. Although aumolertinib demonstrated favorable intracranial efficacy and low rates of any‐grade TRAEs, the lack of high‐grade toxicity data means its safety advantage over drugs like befotertinib (which showed higher toxicity) may be underestimated.

The varying chemical structures among different third‐gen TKIs may result in distinct therapeutic effects and adverse reaction profiles. Aumolertinib replaces the methyl group on the indole ring of osimertinib with a cyclopropyl group. The cyclopropyl group may enhance metabolic stability, potentially reducing the formation of toxic metabolites (e.g., AZ5104) and associated side effects (rash and diarrhea). Furmonertinib replaces the phenyl ring with a pyridyl ring and substitutes the methyl group with a trifluoroethyl group. The pyridyl ring could improve solubility or binding affinity, while the trifluoroethyl group may increase metabolic resistance and prolong drug activity. This might enhance efficacy or reduce dose‐dependent toxicity [29, 30]. Befotertinib's larger molecular weight and broader spectrum of EGFR mutations could contribute to off‐target toxicity, aligning with its higher TRAEs risk [31].

The present study had several limitations. First, it is constrained by the limited availability of data across all outcome measures. Although Bayesian NMA incorporates indirect evidence, the estimates of treatment effects with limited direct comparisons (e.g., rilertinib rely on single trial) may be less stable. Our study provides the most comprehensive indirect comparison of third‐gen EGFR TKIs to date, but its findings should be interpreted alongside emerging evidence from ongoing head‐to‐head trials (e.g., MARIPOSA). Second, even with the restriction to only phase 3 RCTs, variations in trial design and participant heterogeneity may still introduce unmeasured confounders that could potentially bias the estimates. Third, we did not perform transitivity test due to the number of included studies (*n* < 10). All comparisons based on indirect evidence were derived from the common comparator (first‐gen TKIs). Since efficacy and toxicity were consistent across all trials, it is reasonable to infer transitivity. A fourth limitation was the inability to perform subgroup analyses for certain groups potentially associated with TKI efficacy, such as ethnicity or co‐mutations, due to insufficient data.

## 4 Conclusions

In this network meta‐analysis, all third‐gen TKIs outperformed first‐gen TKIs, with no significant differences in efficacy among them for EGFR‐mutated NSCLC in the first‐line setting. Toxicity was generally better tolerated with furmonertinib, aumolertinib, and osimertinib, making them the preferred options when considering both efficacy and safety. These findings can complement the current standard of care for advanced EGFR‐mutated NSCLC.

## 5 Material and Methods

This systematic review and NMA follow the Preferred Reporting Items for Systematic Reviews and Meta‐analyses (PRISMA) guidelines [32] (Table S3). The review protocol for this study was prospectively registered in the Prospective Register of Systematic Reviews (PROSPERO) to ensure transparency, reliability, and novelty (CRD 42024611624). Patients or the public were not involved in the design, conduct, reporting, or dissemination plans of our research.

### 5.1 Search Strategy and Selection Criteria

We conducted a comprehensive search of the Web of Science, Embase, PubMed, Cochrane Library, and ClinicalTrials.gov databases for clinical trials up to November 15, 2024. Our search strategy included a combination of terms such as “NSCLC”, “RCTs”, “EGFR”, “TKIs, osimertinib, aumolertinib, furmonertinib, alflutinib, befotertinib, rezivertinib, rilertinib, lazertinib, and limertinib” (Table S4). To ensure the inclusion of the updated outcomes, we also reviewed abstracts and presentations from major international conferences from 2018 to 2024 including American Society of Clinical Oncology (ASCO) Annual Meeting, European Society for Medical Oncology (ESMO) Congress, World Conference on Lung Cancer (WCLC), and European Lung Cancer Congress (ELCC). Additionally, we manually checked the reference lists of relevant articles and consulted with experts in the field to search for additional studies.

We included published and unpublished phase 3 RCTs that met the following criteria: (1) Trials enrolled treatment‐naive patients with histologically confirmed advanced (stage III/IV/recurrent) NSCLC with EGFR activating mutations; (2) trials that compared any two or more different arms of first‐line third‐gen EGFR‐TKIs treatments for EGFR‐mutated NSCLC patients; and (3) trials that reported on at least one of the following outcomes: OS (defined as the duration from randomization to death due to any cause), PFS (defined as the interval between randomization and the first occurrence of either disease progression or death due to any cause), ORR (defined as the proportion of patients with complete or partial response per RECIST criteria v1.1), intracranial PFS, TRAEs, and severe (grade ≥ 3) TRAEs (assessed by the National Cancer Institute Common Terminology Criteria for Adverse Events). These outcomes were defined in accordance with definitions in the included clinical trials.

Studies that did not meet the inclusion criteria were excluded. Additional exclusion criteria included (1) trials with ambiguous clinical outcomes, (2) trials in which EGFR TKIs were used as neoadjuvant or maintenance treatments, and (3) trials evaluating the cost‐effectiveness effect.

### 5.2 Data Extraction and Risk of Bias Assessment

Two reviewers (W.D. and B.X.) independently conducted data extraction from each eligible study utilizing a pre‐set, standardized form to avoid potential assessment bias by investigators. Any discrepancies were resolved through discussions with other authors (A.L.). Data regarding study and population characteristics (sample size, age, gender, smoking status, Eastern Cooperative Oncology Group Performance Status [ECOG PS], CNS metastases, and EGFR mutational status [exon 19 deletion or L858R]) were extracted. The clinical outcomes extracted included hazard ratios (HRs) with corresponding 95% confidence intervals (95% CIs) for OS and PFS, and the incidence of ORR, any‐grade TRAEs, and TRAEs of grade greater than or equal to 3. We prioritized data evaluated by an independent review committee, adhering to the intention‐to‐treat (ITT) principle. We also considered the most recent data from multiple reports of a single trial. In case of missing data, we reviewed Supporting Information. If the data were still not obtainable, we initiated contact with the corresponding author to request the missing information.

We independently assessed risk of bias of individual studies using the Cochrane Risk of Bias Tool 2.0 [33], which is based on the following categories: random sequence generation (selection bias), allocation concealment (selection bias), blinding of participants and personnel (performance bias), blinding of outcome assessment (detection bias), incomplete outcome data (attrition bias), selective reporting (reporting bias), and other bias. Any disagreements were resolved by consensus and arbitration by a panel of adjudicators.

### 5.3 Data Synthesis and Analysis

The primary outcome was PFS, and secondary outcomes included OS, intracranial PFS, ORR, and TRAEs. The data analysis was conducted using R software (version 4.3.2), within which we used *gemtc* package for the NMA.

Bayesian NMA using Markov chain Monte Carlo simulations was conducted for comparisons between TKIs by synthesizing direct and indirect evidence simultaneously. Bayesian framework is advantageous for its complexity management, allowance for study‐specific covariate inclusion, and its provision of straightforward probabilistic assessments and predictions regarding treatment efficacy [34].

The fixed effects consistency model was applied because the majority of direct evidence originate from one trial [35, 36]. Three different Markov chains were established for running 10,000 burn‐ins and 15,000 sample iterations per chain simultaneously with one step‐size iteration. We evaluated convergence of iterations by visual inspection of the three chains to establish homogeneous parameter estimates and in accordance with the Brooks–Gelman–Rubin diagnostic. Summary estimates were presented as HRs, odds ratios (ORs), or risk ratios (RRs) for the respective outcomes, accompanied by their 95% CrI.

The Bayesian approach was also conducted to estimate the overall rankings of treatments, enabling to rank each outcome from the best to the worst. These rankings were then graphically represented by calculating the surface under the cumulative ranking (SUCRA) value ranging from 0 to 1 [34] based on the ranking profiles of the treatments. Here, 0 means more toxicity and less efficacy compared with 1. Subgroup analyses were also conducted in the NMA to examine the impact of pivotal clinical factors based on various factors such as gender, smoking history, ECOG PS, CNS metastases, and specific EGFR mutations status (exon 19 deletions or L858R).

To evaluate the inconsistency of the model, the node splitting approach was used to define where direct and indirect evidence were separately contrasted on a particular comparison (node) [37, 38]. The assessment of global inconsistency was conducted by comparing the goodness‐of‐fit between the consistency and inconsistency models [37, 39, 40]. A two‐tailed *p*‐value below 0.05 was considered statistically significant.

## Author Contributions

All corresponding and first authors contributed to study concept and design. All authors selected the articles and extracted the data. Li Zhang and Shaodong Hong contacted study investigators and pharmaceutical companies to request additional information. Wei Du, Anlin Li, and Bijing Xiao analyzed and interpreted the data. Wei Du, Anlin Li, and Bijing Xiao wrote the first draft of the report with input from Li Zhang and Shaodong Hong. Yunpeng Yang, Wenfeng Fang, and Yan Huang were responsible for the integrity and accuracy of the data. The corresponding authors attest that all listed authors meet authorship criteria and that no others meeting the criteria have been omitted. All authors have read and approved the final manuscript.

## Ethics Statement

The authors have nothing to report.

## Conflicts of Interest

The authors declare no conflicts of interest.

## Supporting information



Supplemental Table 1. Comparison of the fit goodness between consistency and inconsistency models based on DIC values in network meta‐analysis.
Supplemental Table 2. Node‐splitting analysis of inconsistency.
Supplemental Table 3. PRISMA NMA Checklist of Items to Include When Reporting A Systematic Review Involving a Network Meta‐analysis.
Supplemental Table 4. Literature Search Strategy (october 23rd, 2024).
Supplemental Figure 1. Risk of bias assessments for the included randomized controlled studies according to the Cochrane Risk of Bias Tool 2.0.
Supplemental Figure 2. Bayesian ranking profiles of comparable treatments on efficacy and safety for patients with advanced EGFR mutated NSCLC.
Supplemental Figure 3. Pooled relative PFS of treatment strategies stratified by EGFR mutation in subgroup network meta‐analysis.
Supplemental Figure 4. Pooled relative PFS of treatment strategies stratified by ECOG PS in subgroup network meta‐analysis.
Supplemental Figure 5. Pooled relative PFS of treatment strategies stratified by sex in subgroup network meta‐analysis.
Supplemental Figure 6. Pooled relative PFS of treatment strategies stratified by smoking history in subgroup network meta‐analysis.
Supplemental Figure 7. Pooled relative PFS of treatment strategies stratified by brain metastases in subgroup network meta‐analysis.
Supplemental Figure 8. Forest plot of the pooled estimates: Pairwise vs. Network Meta analyses.
Supplemental Figure 9. Forest plot of the pooled estimates: Pairwise vs. Network Meta analyses.
Supplemental Figure 10. Forest plot of the pooled estimates: Pairwise vs. Network Meta analyses.
Supplemental Figure 11. Convergence of the three chains established by inspection of the history feature and the Brooks‐Gelman‐Rubin diagnostic for objective response rate.
Supplemental Figure 12. Convergence of the three chains established by inspection of the history feature and the Brooks‐Gelman‐Rubin diagnostic for overall survival.
Supplemental Figure 13. Convergence of the three chains established by inspection of the history feature and the Brooks‐Gelman‐Rubin diagnostic for progression‐free survival.
Supplemental Figure 14. Convergence of the three chains established by inspection of the history feature and the Brooks‐Gelman‐Rubin diagnostic for treatment‐related adverse events.

## Data Availability

The data that support the findings of this study are available from the corresponding author upon reasonable request.
