# Supplementary material for: First‐Line Third‐Generation EGFR Tyrosine Kinase Inhibitor Monotherapy for Advanced EGFR‐Mutated Non‐Small Cell Lung Cancer: A Systematic Review and Network Meta‐Analysis
Source: MedComm (2020). 2025 Nov 6;6(11):e70393. doi: 10.1002/mco2.70393 (PMC12592680; doi:10.1002/mco2.70393)

**List of supplementary material**

Supplemental table1. Comparison of the fit goodness between consistency and inconsistency models based on DIC values in network meta-analysis.

Supplemental table2. Node-splitting analysis of inconsistency.

Supplemental table3. PRISMA NMA Checklist of Items to Include When Reporting A Systematic Review Involving a Network Meta-analysis

Supplemental table4. Literature Search Strategy (october 23rd, 2024).

Supplemental figure1. Risk of bias assessments for the included randomized controlled studies according to the Cochrane Risk of Bias Tool 2.0.

Supplemental figure2. Bayesian ranking profiles of comparable treatments on efficacy and safety for patients with advanced EGFR mutated NSCLC.

Supplemental figure3. Pooled relative PFS of treatment strategies stratified by EGFR mutation in subgroup network meta-analysis.

Supplemental figure4. Pooled relative PFS of treatment strategies stratified by ECOG PS in subgroup network meta-analysis.

Supplemental figure5. Pooled relative PFS of treatment strategies stratified by sex in subgroup network meta-analysis

Supplemental figure6. Pooled relative PFS of treatment strategies stratified by smoking history in subgroup network meta-analysis.

Supplemental figure7. Pooled relative PFS of treatment strategies stratified by brain metastases in subgroup network meta-analysis.

Supplemental figure8. Forest plot of the pooled estimates: Pairwise vs. Network Meta analyses.

Supplemental figure9. Forest plot of the pooled estimates: Pairwise vs. Network Meta analyses.

Supplemental figure10. Forest plot of the pooled estimates: Pairwise vs. Network Meta analyses.

Supplemental figure11. Convergence of the three chains established by inspection of the history feature and the Brooks-Gelman-Rubin diagnostic for objective response rate.

Supplemental figure12. Convergence of the three chains established by inspection of the history feature and the Brooks-Gelman-Rubin diagnostic for overall survival.

Supplemental figure13. Convergence of the three chains established by inspection of the history feature and the Brooks-Gelman-Rubin diagnostic for progression-free survival.

Supplemental figure14. Convergence of the three chains established by inspection of the history feature and the Brooks-Gelman-Rubin diagnostic for treatment-related adverse events.

**Supplemental Table 1. Comparison of the fit goodness between consistency and inconsistency models based on DIC values in network meta-analysis.**

| **Outcome** | **Model** | **DIC** | **I^2^(%)** |
| --- | --- | --- | --- |
| PFS | Consistency | 12.25 | 4 |
|  | Inconsistency | 14.00 | 14 |
| OS | Consistency | 8.08 | 2 |
|  | Inconsistency | 10.00 | 20 |
| intracranial PFS | Consistency | 10 | 20 |
|  | Inconsistency | 10 | 20 |
| ORR | Consistency | 26.37 | 3 |
|  | Inconsistency | 28.10 | 7 |
| Intracranial ORR | Consistency | 21.28 | 15 |
|  | Inconsistency | 21.30 | 16 |
| TRAE | Consistency | 19.57 | 11 |
|  | Inconsistency | 19.56 | 11 |
| grade≥3 TREA | Consistency | 20.06 | 10 |
|  | Inconsistency | 20.09 | 10 |

The DIC provides a measure of model fit adjusted with the complexity of the model, with lower values correspond to preferable models and differences of 5 considered important.

Abbreviations: DIC, Deviance information criterion; PFS, progression free survival; OS, overall survival; ORR, objective response rate; TRAE, treatment-related adverse events

**Supplemental Table 2. Node-splitting analysis of inconsistency.**

| **Nodes** | **Direct effect** | **Indirect effect** | **Overall** | ***P*** |
| --- | --- | --- | --- | --- |
| **Progression-free survival** | | | | |
| lazertinib vs 1st TKI | 0.73 (0.65, 0.82) | 0.70 (0.61, 0.80) | 0.72 (0.66, 0.79) | 0.62 |
| osimertinib vs lazertinib | 1.00 (0.92, 1.10) | 0.96 (0.83, 1.10) | 1.00 (0.92, 1.10) | 0.61 |
| osimertinib vs 1st TKI | 0.71 (0.64, 0.78) | 0.74 (0.64, 0.86) | 0.72 (0.66, 0.78) | 0.62 |
| **Overall survival** | | | | |
| lazertinib vs 1st TKI | 0.88 (0.75, 1.00) | 0.91 (0.77, 1.10) | 0.89 (0.79, 1.00) | 0.78 |
| osimertinib vs lazertinib | 1.00 (0.87, 1.10) | 1.00 (0.86, 1.20) | 1.00 (0.90, 1.10) | 0.78 |
| osimertinib vs 1st TKI | 0.91 (0.82, 1.00) | 0.88 (0.71, 1.10) | 0.90 (0.83, 0.99) | 0.78 |
| **Objective response rate** | | | | |
| lazertinib vs 1st TKI | 0.89 (0.47, 1.69) | 1.12 (0.47, 2.67) | 0.96(0.59, 1.59) | 0.64 |
| osimertinib vs lazertinib | 1.13 (0.60, 2.12) | 1.45 (0.60, 3.41) | 1.24 (0.76, 1.99) | 0.63 |
| osimertinib vs 1st TKI | 1.28 (0.70, 2.33) | 1.02 (0.42, 2.47) | 1.19 (0.74, 1.91) | 0.65 |

Significant values (*P* ≤ 0.05) indicating a significant inconsistency between the direct effect and indirect effects.

Only comparisons involving two or more trials are presented here.

Abbreviations: TKIs, tyrosine kinase inhibitors.

**Supplemental Table3. PRISMA NMA Checklist of Items to Include When Reporting A Systematic Review Involving a Network Meta-analysis**

| **Section/Topic** | **Item #** | **Checklist Item** | **Reported on Page #** |
| --- | --- | --- | --- |
| **TITLE** |  |  |  |
| Title | 1 | Identify the report as a systematic review *incorporating a network meta-analysis (or related form of meta-analysis).* | 1 |
| **ABSTRACT** |  |  |  |
| Structured summary | 2 | Provide a structured summary including, as applicable:  **Background:** main objectives  **Methods:** data sources; study eligibility criteria, participants, and interventions; study appraisal; and *synthesis methods, such as network meta-analysis.*  **Results:** number of studies and participants identified; summary estimates with corresponding confidence/credible intervals; *treatment rankings may also be discussed. Authors may choose to summarize pairwise comparisons against a chosen treatment included in their analyses for brevity.*  **Discussion/Conclusions:** limitations; conclusions and implications of findings.  **Other:** primary source of funding; systematic review registration number with registry name. | 2 |
| **INTRODUCTION** |  |  |  |
| Rationale | 3 | Describe the rationale for the review in the context of what is already known*, including mention of why a network meta-analysis has been conducted.* | ***3-4*** |
| Objectives | 4 | Provide an explicit statement of questions being addressed, with reference to participants, interventions, comparisons, outcomes, and study design (PICOS). | 4 |
| **METHODS** |  |  |  |
| Protocol and registration | 5 | Indicate whether a review protocol exists and if and where it can be accessed (e.g., Web address); and, if available, provide registration information, including registration number. | 5 |
| Eligibility criteria | 6 | Specify study characteristics (e.g., PICOS, length of follow-up) and report characteristics (e.g., years considered, language, publication status) used as criteria for eligibility, giving rationale. *Clearly describe eligible treatments included in the treatment network, and note whether any have been clustered or merged into the same node (with justification).* | ***5-6*** |
| Information sources | 7 | Describe all information sources (e.g., databases with dates of coverage, contact with study authors to identify additional studies) in the search and date last searched. | 5 |
| Search | 8 | Present full electronic search strategy for at least one database, including any limits used, such that it could be repeated. | 5 |
| Study selection | 9 | State the process for selecting studies (i.e., screening, eligibility, included in systematic review, and, if applicable, included in the meta-analysis). | 5-6 |
| Data collection process | 10 | Describe method of data extraction from reports (e.g., piloted forms, independently, in duplicate) and any processes for obtaining and confirming data from investigators. | 6-7 |
| Data items | 11 | List and define all variables for which data were sought (e.g., PICOS, funding sources) and any assumptions and simplifications made. | 6-7 |
| Geometry of the network | **S1** | Describe methods used to explore the geometry of the treatment network under study and potential biases related to it. This should include how the evidence base has been graphically summarized for presentation, and what characteristics were compiled and used to describe the evidence base to readers. | ***10*** |
| Risk of bias within individual studies | 12 | Describe methods used for assessing risk of bias of individual studies (including specification of whether this was done at the study or outcome level), and how this information is to be used in any data synthesis. | 7 |
| Summary measures | 13 | State the principal summary measures (e.g., risk ratio, difference in means). *Also describe the use of additional summary measures assessed, such as treatment rankings and surface under the cumulative ranking curve (SUCRA) values, as well as modified approaches used to present summary findings from meta-analyses.* | 7 |
| Planned methods of analysis | 14 | Describe the methods of handling data and combining results of studies for each network meta-analysis. This should include, but not be limited to:   - *Handling of multi-arm trials;* - *Selection of variance structure;* - *Selection of prior distributions in Bayesian analyses; and* - *Assessment of model fit.* | 7-8 |
| Assessment of Inconsistency | S2 | Describe the statistical methods used to evaluate the agreement of direct and indirect evidence in the treatment network(s) studied. Describe efforts taken to address its presence when found. | 8-9 |
| Risk of bias across studies | 15 | Specify any assessment of risk of bias that may affect the cumulative evidence (e.g., publication bias, selective reporting within studies). | **7** |
| Additional analyses | 16 | Describe methods of additional analyses if done, indicating which were pre-specified. This may include, but not be limited to, the following:   - Sensitivity or subgroup analyses; - Meta-regression analyses; - *Alternative formulations of the treatment network; and* - *Use of alternative prior distributions for Bayesian analyses (if applicable).* | ***8-9*** |
| **RESULTS†** |  |  |  |
| Study selection | 17 | Give numbers of studies screened, assessed for eligibility, and included in the review, with reasons for exclusions at each stage, ideally with a flow diagram. | Figure 1 |
| Presentation of network structure | **S3** | Provide a network graph of the included studies to enable visualization of the geometry of the treatment network. | ***Figure2*** |
| Summary of network geometry | **S4** | Provide a brief overview of characteristics of the treatment network. This may include commentary on the abundance of trials and randomized patients for the different interventions and pairwise comparisons in the network, gaps of evidence in the treatment network, and potential biases reflected by the network structure. | ***10*** |
| Study characteristics | 18 | For each study, present characteristics for which data were extracted (e.g., study size, PICOS, follow-up period) and provide the citations. | ***10,TableS3*** |
| Risk of bias within studies | 19 | Present data on risk of bias of each study and, if available, any outcome level assessment. | 10 |
| Results of individual studies | 20 | For all outcomes considered (benefits or harms), present, for each study: 1) simple summary data for each intervention group, and 2) effect estimates and confidence intervals. *Modified approaches may be needed to deal with information from larger networks.* | ***10-14*** |
| Synthesis of results | 21 | Present results of each meta-analysis done, including confidence/credible intervals. *In larger networks, authors may focus on comparisons versus a particular comparator (e.g. placebo or standard care), with full findings presented in an appendix. League tables and forest plots may be considered to summarize pairwise comparisons.* If additional summary measures were explored (such as treatment rankings), these should also be presented. | ***10-14*** |
| Exploration for inconsistency | S5 | Describe results from investigations of inconsistency. This may include such information as measures of model fit to compare consistency and inconsistency models, *P* values from statistical tests, or summary of inconsistency estimates from different parts of the treatment network. | ***14*** |
| Risk of bias across studies | 22 | Present results of any assessment of risk of bias across studies for the evidence base being studied. | 10 |
| Results of additional analyses | 23 | Give results of additional analyses, if done (e.g., sensitivity or subgroup analyses, meta-regression analyses*, alternative network geometries studied, alternative choice of prior distributions for Bayesian analyses,* and so forth). | ***14*** |
| **DISCUSSION** |  |  |  |
| Summary of evidence | 24 | Summarize the main findings, including the strength of evidence for each main outcome; consider their relevance to key groups (e.g., healthcare providers, users, and policy-makers). | 15-17 |
| Limitations | 25 | Discuss limitations at study and outcome level (e.g., risk of bias), and at review level (e.g., incomplete retrieval of identified research, reporting bias). *Comment on the validity of the assumptions, such as transitivity and consistency. Comment on any concerns regarding network geometry (e.g., avoidance of certain comparisons).* | 18 |
| Conclusions | 26 | Provide a general interpretation of the results in the context of other evidence, and implications for future research. | 19 |
| **FUNDING** |  |  |  |
| Funding | 27 | Describe sources of funding for the systematic review and other support (e.g., supply of data); role of funders for the systematic review. This should also include information regarding whether funding has been received from manufacturers of treatments in the network and/or whether some of the authors are content experts with professional conflicts of interest that could affect use of treatments in the network. | ***20*** |

PICOS = population, intervention, comparators, outcomes, study design.

* Text in italics indicateS wording specific to reporting of network meta-analyses that has been added to guidance from the PRISMA statement.

† Authors may wish to plan for use of appendices to present all relevant information in full detail for items in this section.

**Supplemental Table 4. Literature Search Strategy (october 23rd, 2024).**

| **ID** | **Search** | **Hits** |
| --- | --- | --- |
| **PubMed** | | |
| 1 | (non small cell lung carcinoma [Title/Abstract]) OR (nsclc[Title/Abstract]) | 69773 |
| 2 | (epidermal growth factor receptor [Title/Abstract]) OR (EGFR[Title/Abstract]) | 113267 |
| 3 | (((Lazertinib[Title/Abstract]) OR (Limertinib[Title/Abstract])) OR (SH-1028[Title/Abstract])) OR ((((((((((tyrosine kinase inhibitor[Title/Abstract]) OR (TKI[Title/Abstract])) OR (Third generation[Title/Abstract])) OR (osimertinib[Title/Abstract])) OR (almonertinib[Title/Abstract])) OR (furmonertinib[Title/Abstract])) OR (alflutinib[Title/Abstract])) OR (befotertinib[Title/Abstract])) OR (rezivertinib[Title/Abstract])) OR (Rilertinib[Title/Abstract])) | 50235 |
| 4 | ((first line [Title/Abstract]) OR (Treatment naïve [Title/Abstract])) OR (untreated[Title/Abstract]) | 337754 |
| 5 | (((((Randomized Controlled Trial [Title/Abstract]) OR (controlled clinical trial [Title/Abstract])) OR (randomized [Title/Abstract])) OR (randomly[Title/Abstract])) OR (trial[Title/Abstract])) OR (phase[Title/Abstract]) | 2356431 |
| 6 | English [Language] | 32995096 |
| 7 | ("0001/01/01"[Date - Publication]: "2024/11/15"[Date - Publication]) | 37854344 |
| 8 | (((((((non small cell lung carcinoma[Title/Abstract]) OR (nsclc[Title/Abstract])) AND ((epidermal growth factor receptor[Title/Abstract]) OR (EGFR[Title/Abstract]))) AND ((((Lazertinib[Title/Abstract]) OR (Limertinib[Title/Abstract])) OR (SH-1028[Title/Abstract])) OR ((((((((((tyrosine kinase inhibitor[Title/Abstract]) OR (TKI[Title/Abstract])) OR (Third generation[Title/Abstract])) OR (osimertinib[Title/Abstract])) OR (almonertinib[Title/Abstract])) OR (furmonertinib[Title/Abstract])) OR (alflutinib[Title/Abstract])) OR (befotertinib[Title/Abstract])) OR (rezivertinib[Title/Abstract])) OR (Rilertinib[Title/Abstract])))) AND (((first line[Title/Abstract]) OR (Treatment naive[Title/Abstract])) OR (untreated[Title/Abstract]))) AND ((((((Randomized Controlled Trial[Title/Abstract]) OR (controlled clinical trial[Title/Abstract])) OR (randomised[Title/Abstract])) OR (randomly[Title/Abstract])) OR (trial[Title/Abstract])) OR (phase[Title/Abstract]))) AND (english[Language])) AND (("0001/01/01"[Date - Publication] : "2024/11/15"[Date - Publication])) | 430 |
| **Embase** | | |
| #1 | 'non small cell lung cancer’: ab,ti OR 'nsclc':ab,ti | 158110 |
| #2 | 'epidermal growth factor receptor’: ab,ti OR 'egfr':ab,ti | 196297 |
| #3 | ('tyrosine kinase inhibitor’: ab,ti OR 'kinase inhibitor':ab,ti OR 'tki':ab,ti OR third) AND generation:ab,ti OR osimertinib:ab,ti OR almonertinib:ab,ti OR furmonertinib:ab,ti OR alflutinib:ab,ti OR befotertinib:ab,ti OR rezivertinib:ab,ti OR rilertinib:ab,ti OR lazertinib:ab,ti OR limertinib:ab,ti OR 'sh 1028':ab,ti | 57755 |
| #4 | (first AND line:ab,ti OR treatment) AND naive:ab,ti OR untreated:ab,ti | 383133 |
| #5 | [clinical study]/lim OR [controlled clinical trial]/lim OR [randomized controlled trial]/lim | 13062572 |
| #6 | [01-01-0001]/sd NOT [11-15-2024]/sd | 45791369 |
| #7 | #1 AND #2 AND #3 AND #4 AND #5 AND #6 | 512 |
| **Web of Science** | | |
| #1 | (AB= (non small cell lung carcinoma)) OR AB=(nsclc) | 99006 |
| #2 | ((AB= (first line)) OR AB= (treatment naïve)) OR AB=(untreated) | 1870904 |
| #3 | ((((AB= (Randomized Controlled Trial)) OR AB=(randomised)) OR AB=(randomly)) OR AB=(trial)) OR AB=(phase) | 9866314 |
| #4 | ALL= ("epidermal growth factor receptor" OR "EGFR") | 182843 |
| #5 | (((((((((((((AB= (tyrosine kinase inhibitor)) OR AB=(TKI)) OR AB=(Third generation)) OR AB=(osimertinib)) OR AB=(almonertinib)) OR AB=(furmonertinib)) OR AB=(alflutinib)) OR AB=(befotertinib)) OR AB=(rezivertinib)) OR AB=(Rilertinib)) OR AB=(Lazertinib)) OR AB=(Limertinib)) OR AB=(Sh-1028)) | 274429 |
| #6 | DOP= (1900-01-01/2024-11-15) | 177393061 |
| #7 | #6 AND #5 AND #4 AND #3 AND #2 AND #1 | 872 |
| **Cochrane Library** | | |
| #1MeSH descriptor: [Carcinoma, Non-Small-Cell Lung] this term only  #2(alflutinib): ti,ab,kw OR (befotertinib):ti,ab,kw OR (Rilertinib):ti,ab,kw OR (rezivertinib):ti,ab,kw OR (furmonertinib):ti,ab,kw  #3(first line): ti,ab,kw OR (treatmentnaïve):ti,ab,kw OR (untreated):ti,ab,kw  #4(Randomized Controlled Trial): ti,ab,kw OR (phase):ti,ab,kw  #5(almonertinib): ti,ab,kw OR (osimertinib):ti,ab,kw OR (Third generation):ti,ab,kw OR (TKI):ti,ab,kw OR (tyrosine kinase inhibitor):ti,ab,kw  #6(Lazertinib): ti,ab,kw OR (Limertinib):ti,ab,kw OR (Sh-1028):ti,ab,kw  #7#2 OR #5 OR #6  #8#1 AND #3 AND #4 AND #7 in Trials | | 191 |
| **ClinicalTrials.gov** | | |
| "epidermal growth factor receptor" OR "EGFR" \| Interventional Studies \| "carcinoma, non small cell lung" OR "non small cell lung cancer" OR "NSCLC" \| \| Other terms: first line OR Treatment naive OR untreated \| "tyrosine kinase inhibitor" OR "kinase inhibitor" OR "TKI" \| Active, not recruiting, Completed studies \| Interventional studies | | 228 |

**Supplemental Figure 1 Risk of bias assessments for the included randomized controlled studies according to the Cochrane Risk of Bias Tool 2.0.**


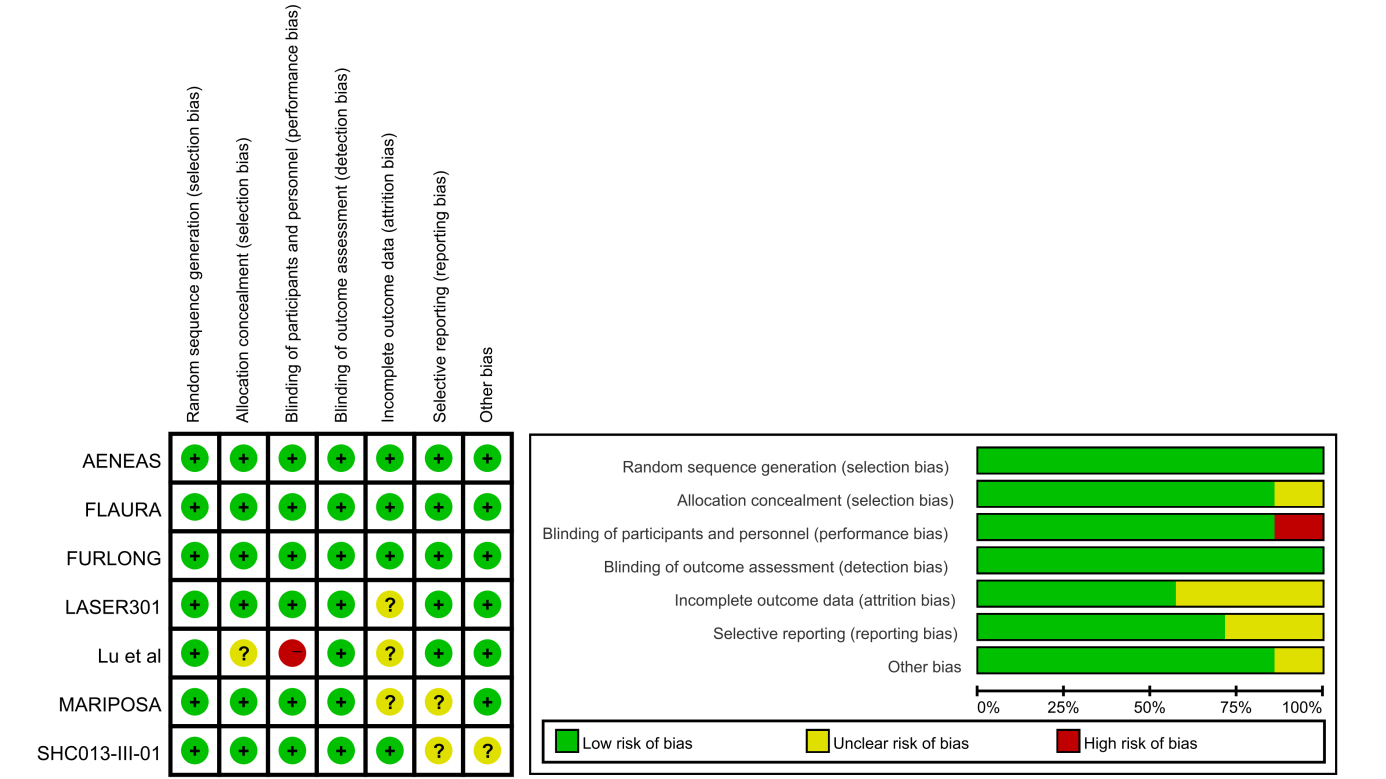


**Supplemental Figure 2 Bayesian ranking profiles of comparable treatments on efficacy and safety for patients with advanced EGFR mutated NSCLC.**


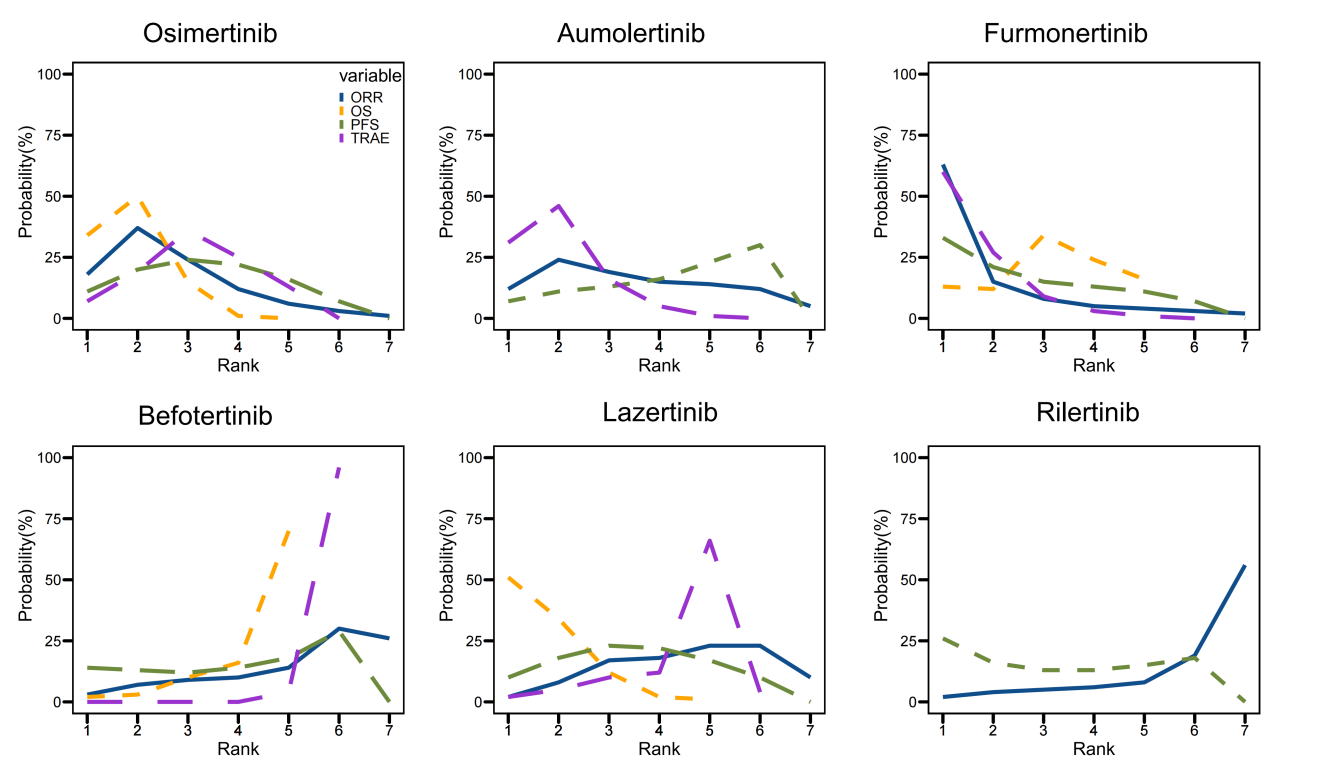


Profiles indicate the probability of each comparable treatment being ranked from first to last on PFS ^a^, OS ^a^, ORR ^b^, and TRAE ^a^. a: Those with lower values ranked first.b: Those with higher values ranked first.Abbreviations: EGFR, epidermal growth factor receptor; NSCLC, non-small cell lung cancer; OS, overall survival; PFS, progression free survival; ORR, objective response rate; TRAE, treatment-related adverse event.

**Supplemental Figure 3. Pooled relative PFS of treatment strategies stratified by EGFR mutation in subgroup network meta-analysis.**


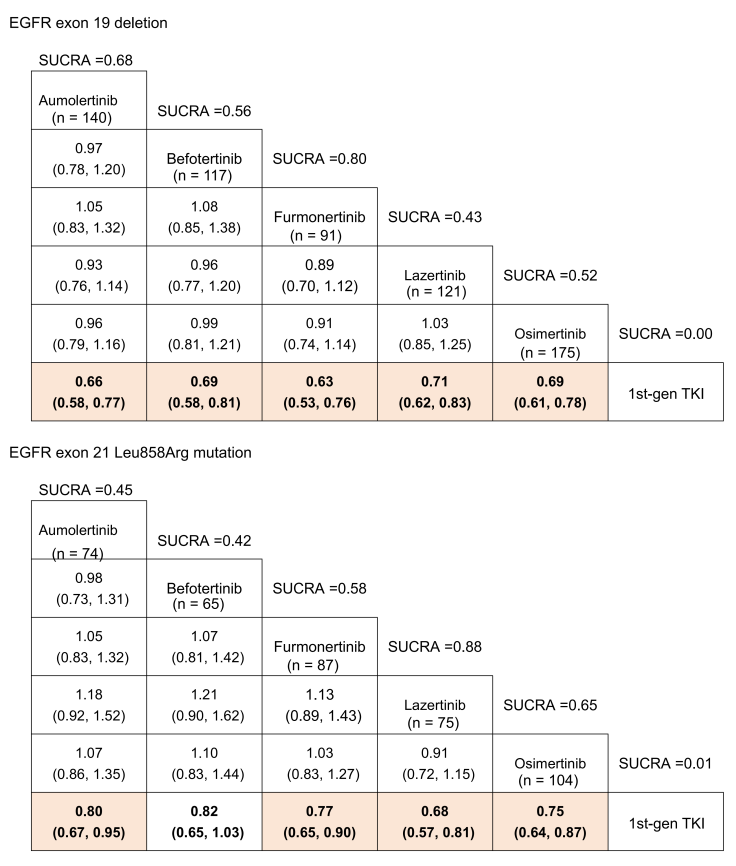


Numbers in each cell represent pooled hazard ratios for comparison of the column-defining treatment versus the row-defining treatment, along with their corresponding 95% credible intervals. Numbers upon the treatments represent SUCRA ranking values. Significant results are highlighted in color.

Abbreviations: PFS, progression free survival; EGFR, epidermal growth factor receptor; SUCRA, surface under the cumulative ranking.

**Supplemental Figure 4. Pooled relative PFS of treatment strategies stratified by ECOG PS in subgroup network meta-analysis.**


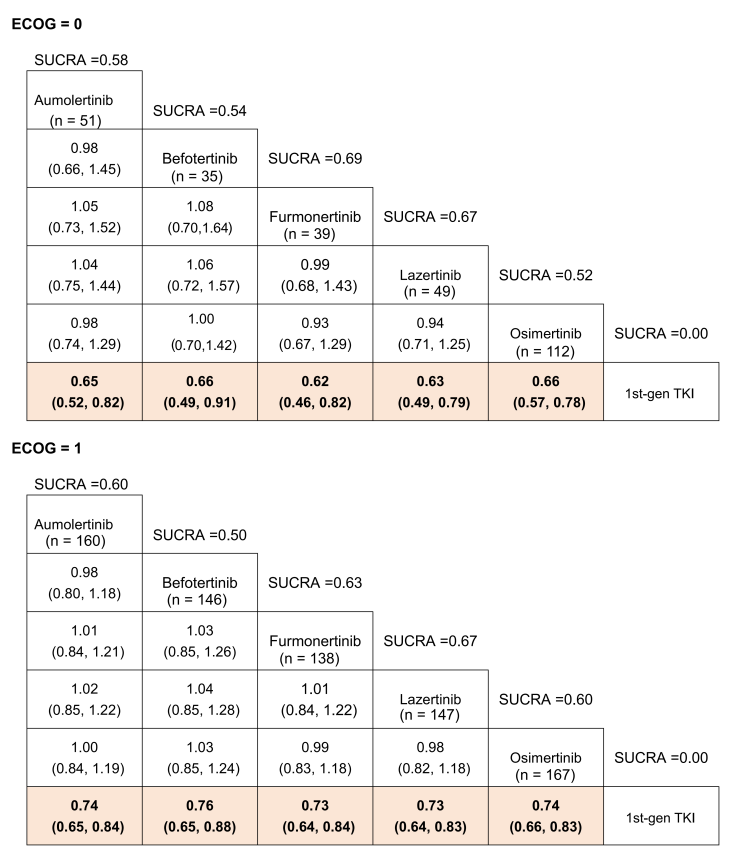


Numbers in each cell represent pooled hazard ratios for comparison of the column-defining treatment versus the row-defining treatment, along with their corresponding 95% credible intervals. Numbers upon the treatments represent SUCRA ranking values. Significant results are highlighted in color.

Abbreviations: PFS, progression free survival; ECOG PS, Eastern Cooperative Oncology Group Performance Status; SUCRA, surface under the cumulative ranking.

**Supplemental Figure 5. Pooled relative PFS of treatment strategies stratified by sex in subgroup network meta-analysis.**


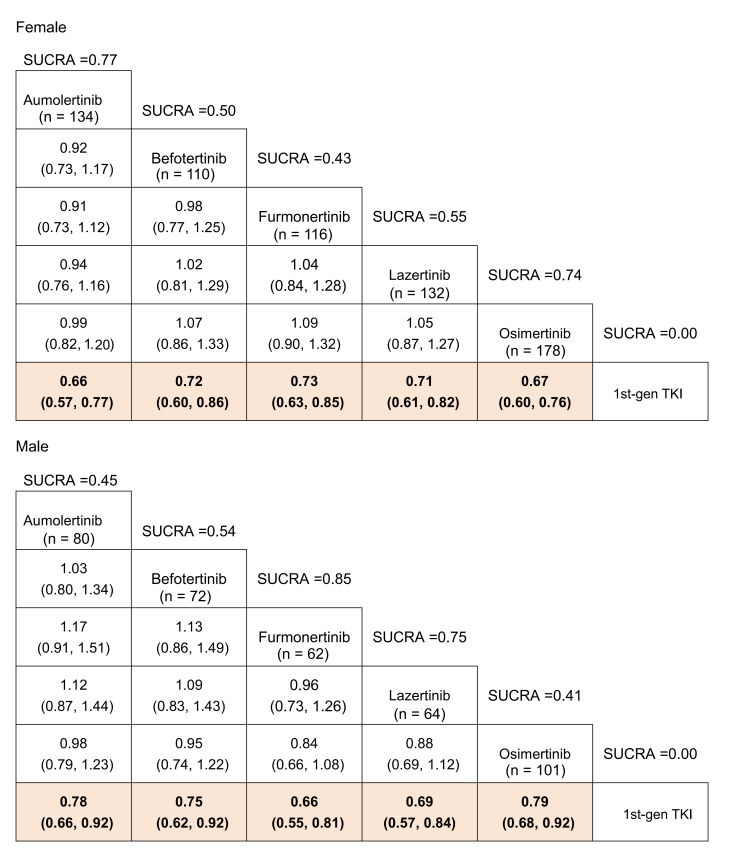


Numbers in each cell represent pooled hazard ratios for comparison of the column-defining treatment versus the row-defining treatment, along with their corresponding 95% credible intervals. Numbers upon the treatments represent SUCRA ranking values. Significant results are highlighted in color.

Abbreviations: PFS, progression free survival; SUCRA, surface under the cumulative ranking.

**Supplemental Figure 6. Pooled relative PFS of treatment strategies stratified by smoking history in subgroup network meta-analysis.**


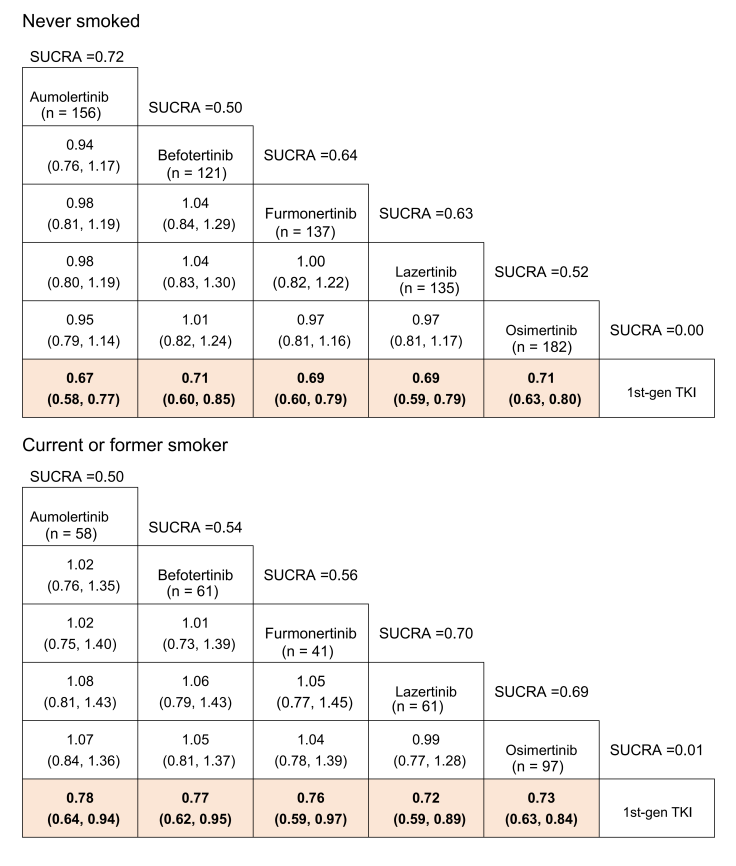


Numbers in each cell represent pooled hazard ratios for comparison of the column-defining treatment versus the row-defining treatment, along with their corresponding 95% credible intervals. Numbers upon the treatments represent SUCRA ranking values. Significant results are highlighted in color.

Abbreviations: PFS, progression free survival; SUCRA, surface under the cumulative ranking.

**Supplemental Figure 7. Pooled relative PFS of treatment strategies stratified by brain metastases in subgroup network meta-analysis.**


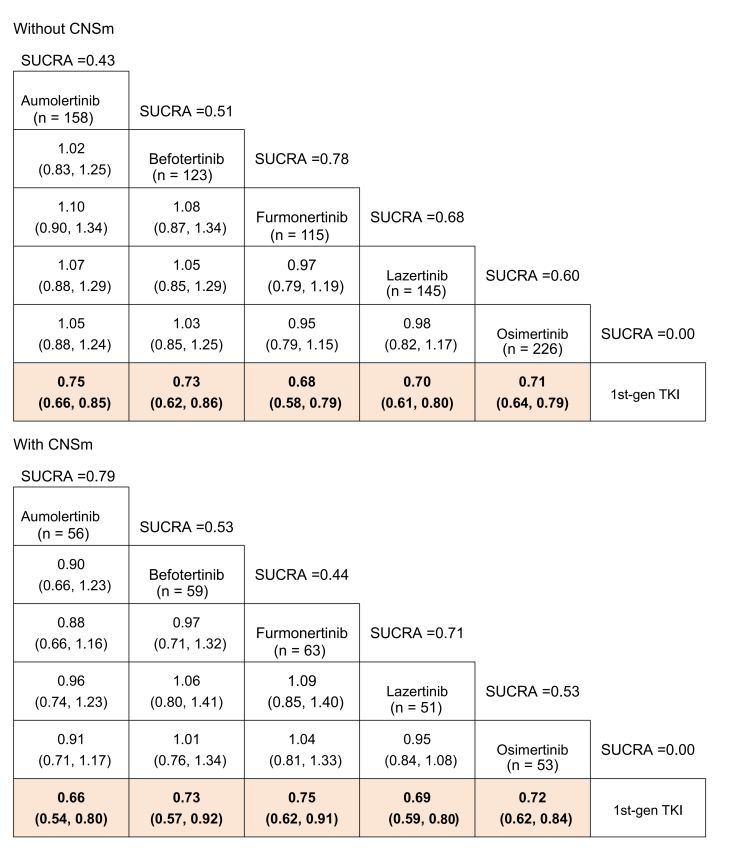


Numbers in each cell represent pooled hazard ratios for comparison of the column-defining treatment versus the row-defining treatment, along with their corresponding 95% credible intervals. Numbers upon the treatments represent SUCRA ranking values. Significant results are highlighted in color.

Abbreviations: PFS, progression free survival; SUCRA, surface under the cumulative ranking;CNSm,central nervous system metastasis.

**Supplemental Figure 8. Forest plot of the pooled estimates: Pairwise vs. Network Meta analyses.**


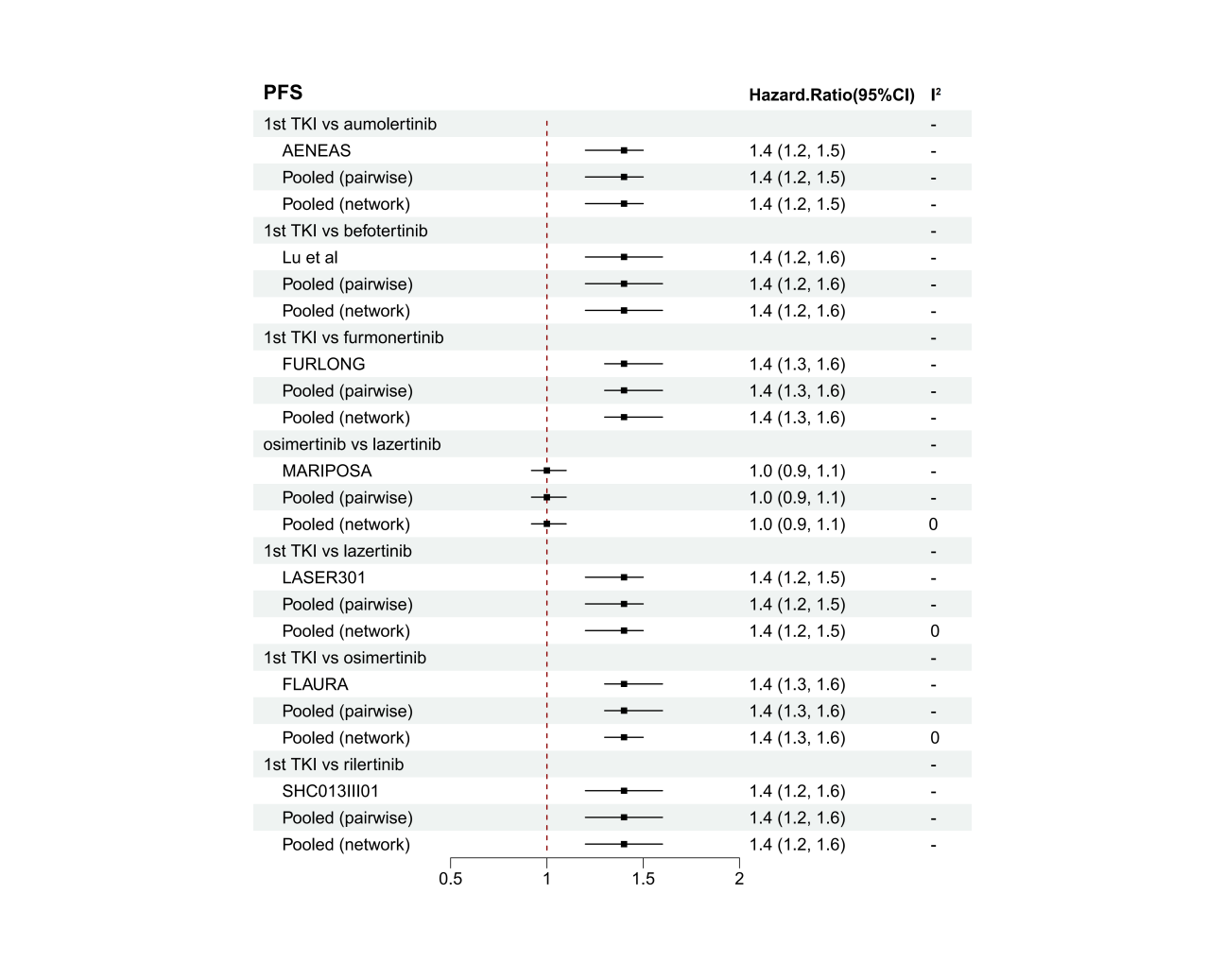


Abbreviations: PFS, progression-free survival; TKI, tyrosine kinase inhibitor.

**Supplemental Figure 9. Forest plot of the pooled estimates: Pairwise vs. Network Meta analyses.**


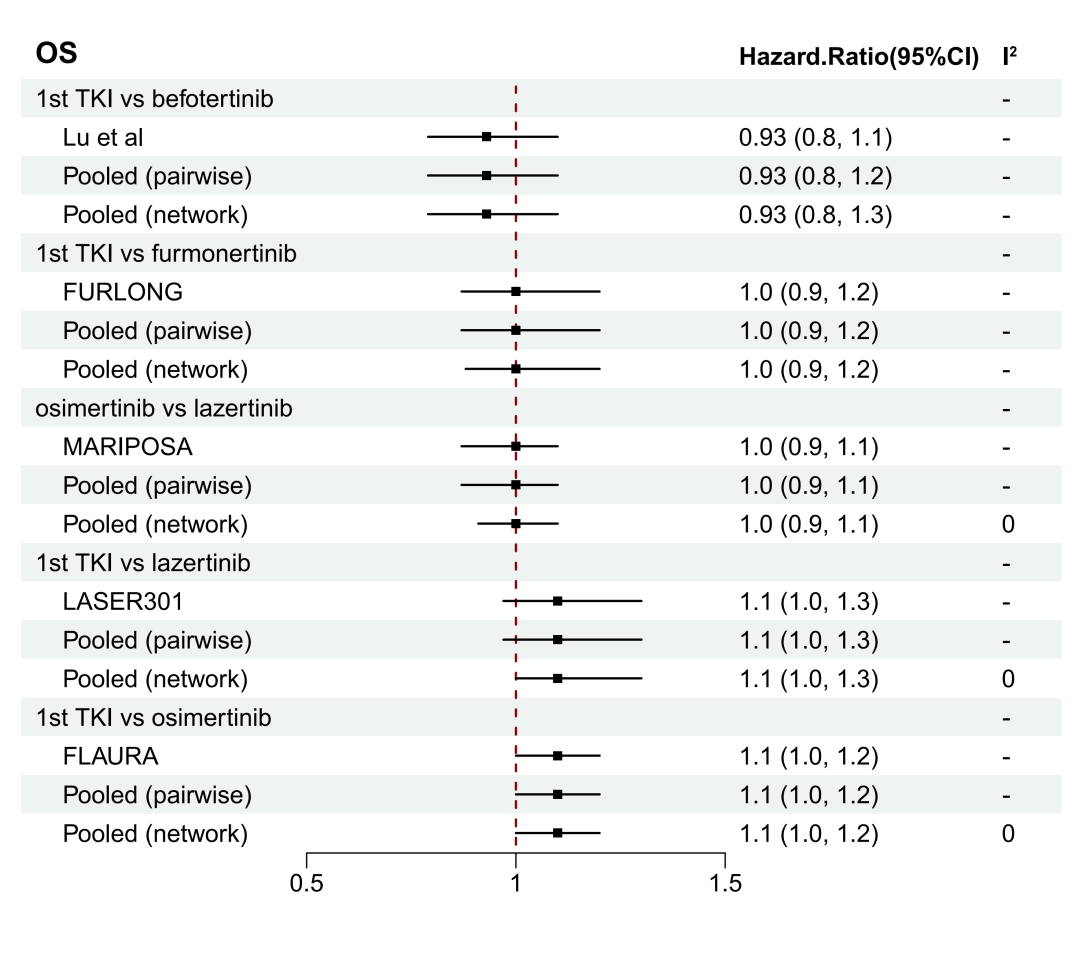


Abbreviations: OS, overall survival; TKI, tyrosine kinase inhibitor.

**Supplemental Figure 10. Forest plot of the pooled estimates: Pairwise vs. Network Meta analyses.**


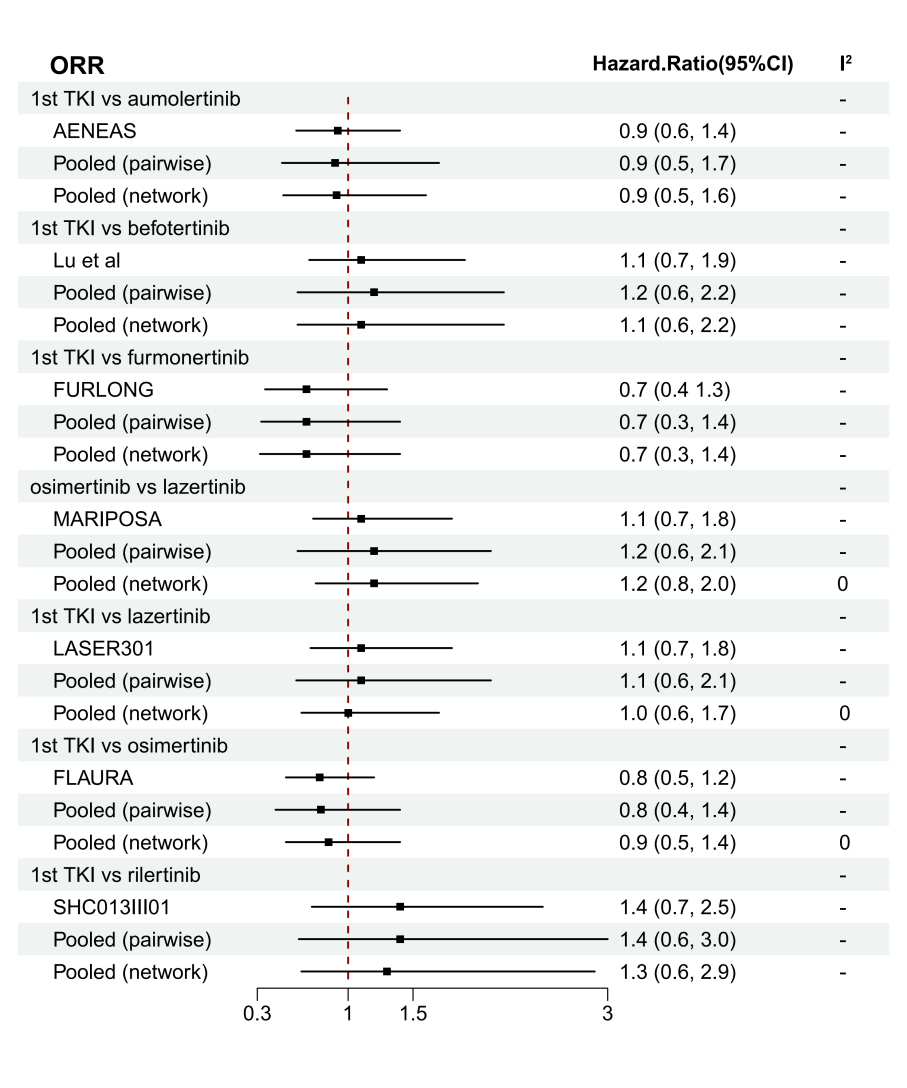


Abbreviations: ORR, objective response rate, TKI, tyrosine kinase inhibitor.

**Supplemental Figure 11. Convergence of the three chains established by inspection of the history feature and the Brooks-Gelman-Rubin diagnostic for objective response rate.**


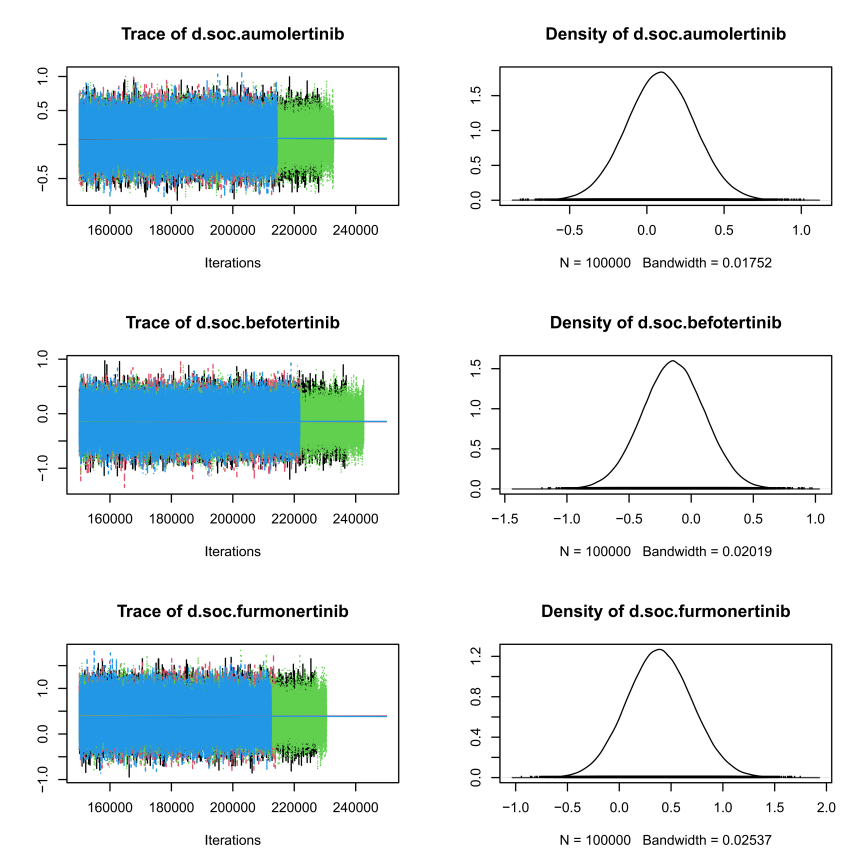


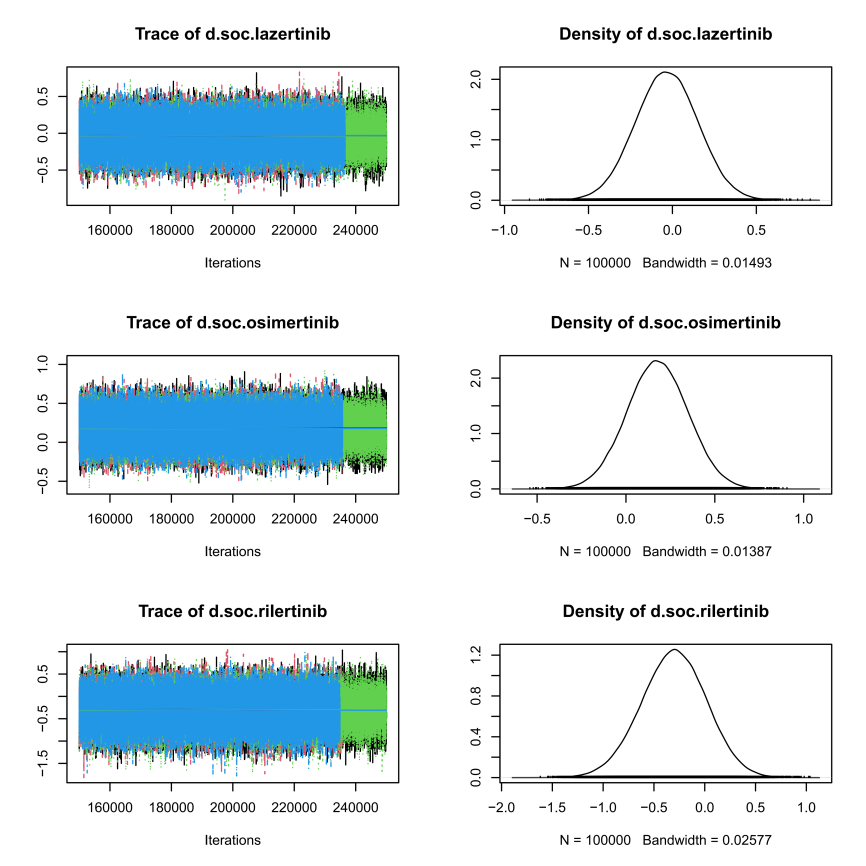


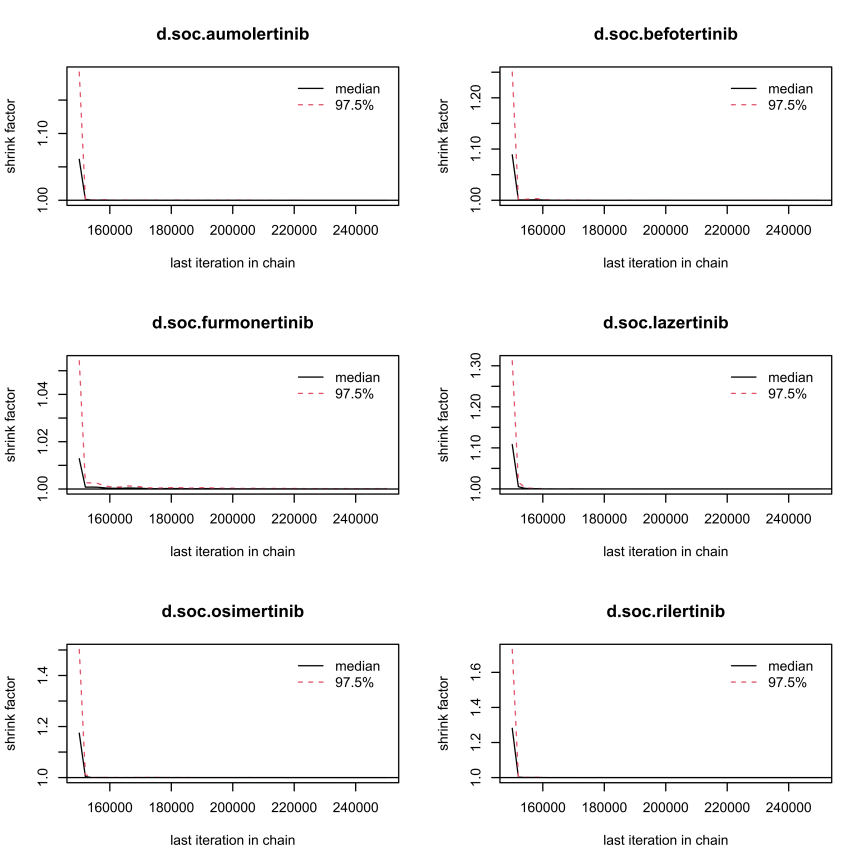


**Supplemental Figure 12.Convergence of the three chains established by inspection of the history feature and the Brooks-Gelman-Rubin diagnostic for overall survival.**


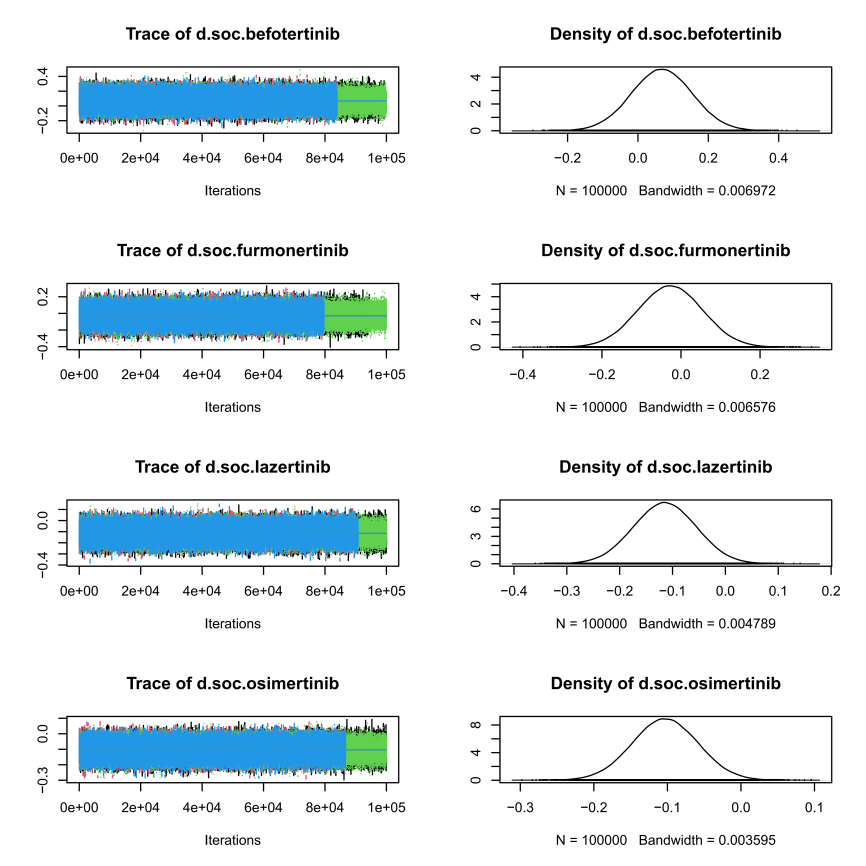


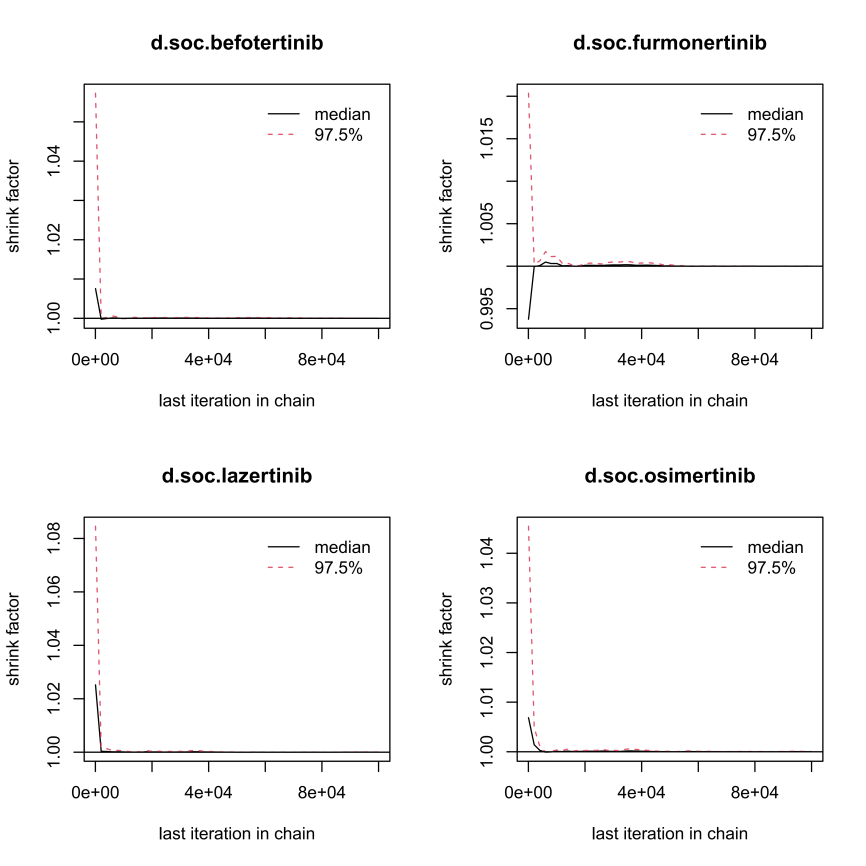


**Supplemental Figure 13.Convergence of the three chains established by inspection of the history feature and the Brooks-Gelman-Rubin diagnostic for progression-free survival.**


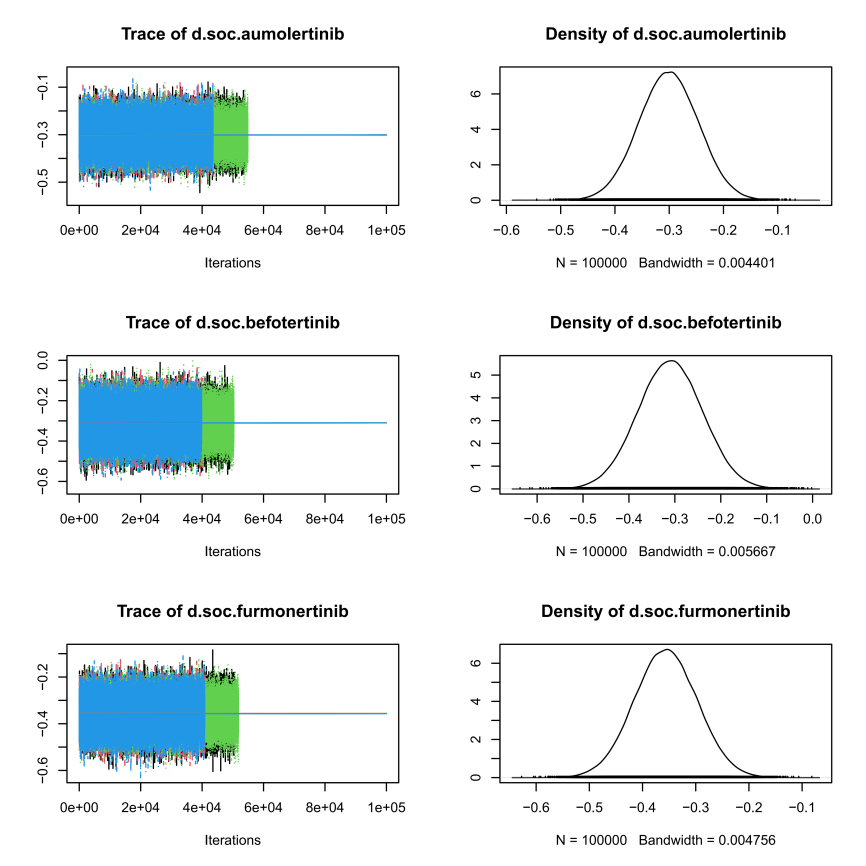


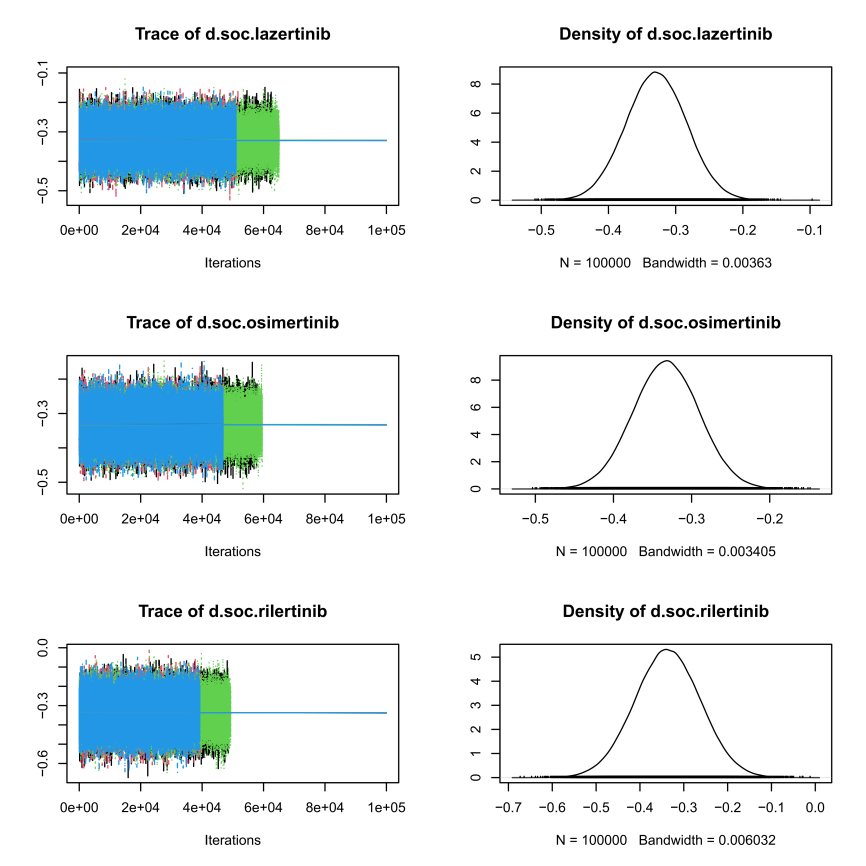


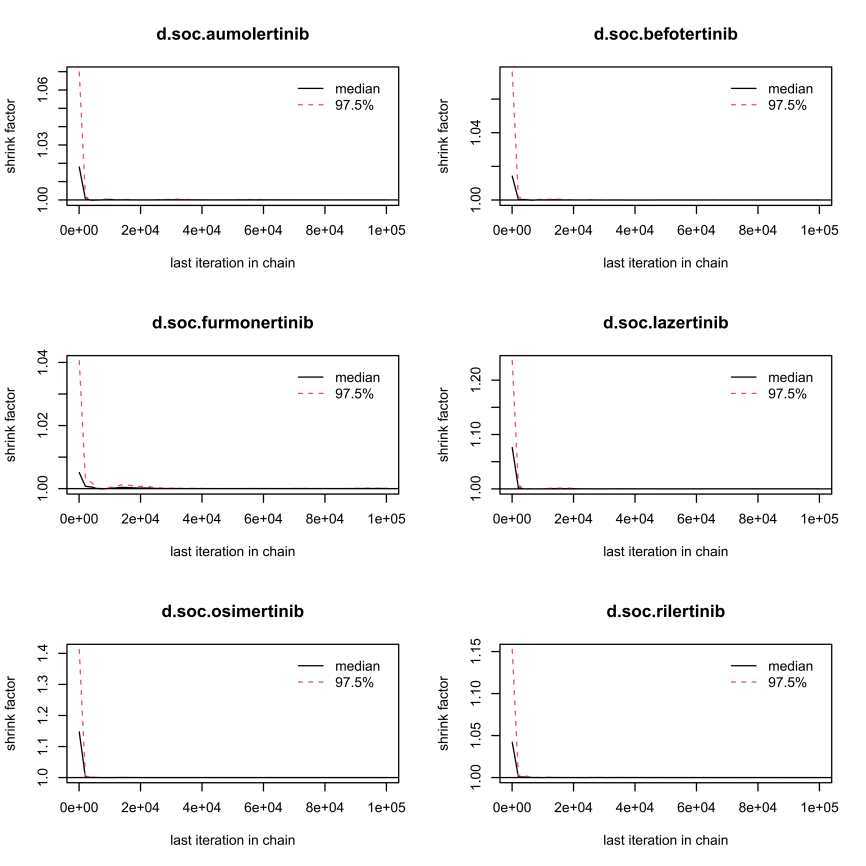


**Supplemental Figure 14.Convergence of the three chains established by inspection of the history feature and the Brooks-Gelman-Rubin diagnostic for treatment-related adverse events.**


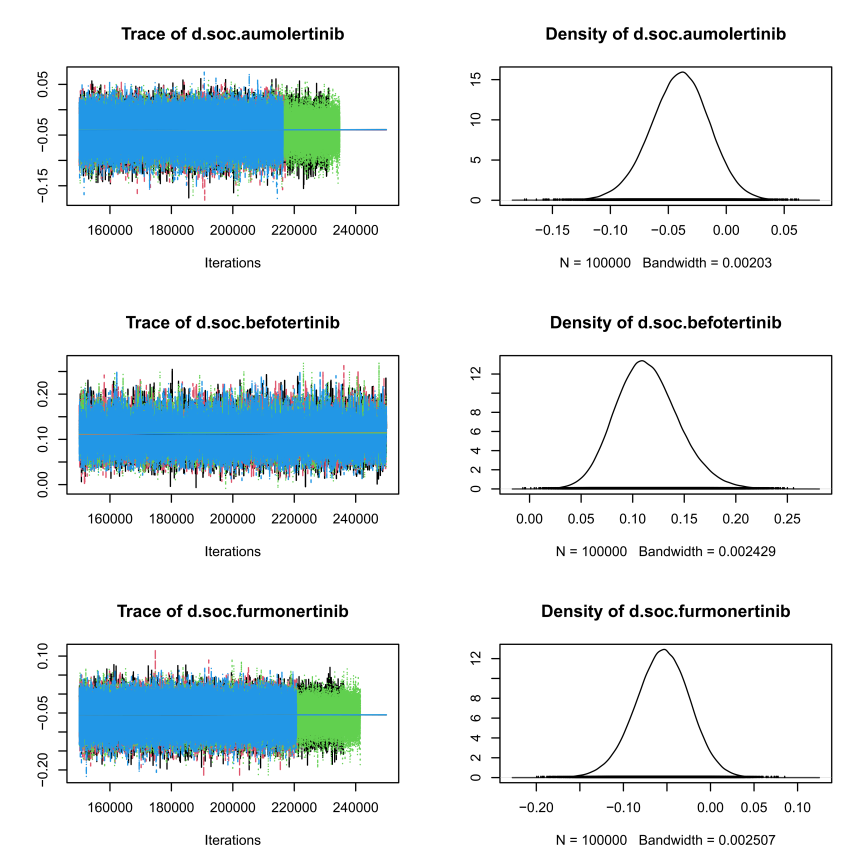


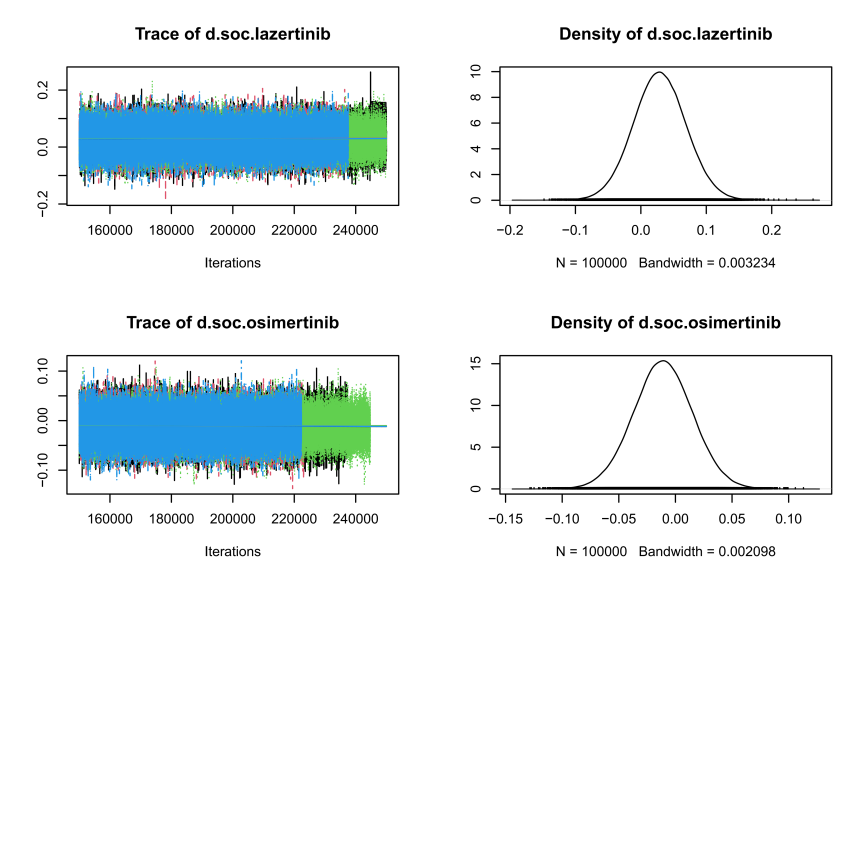


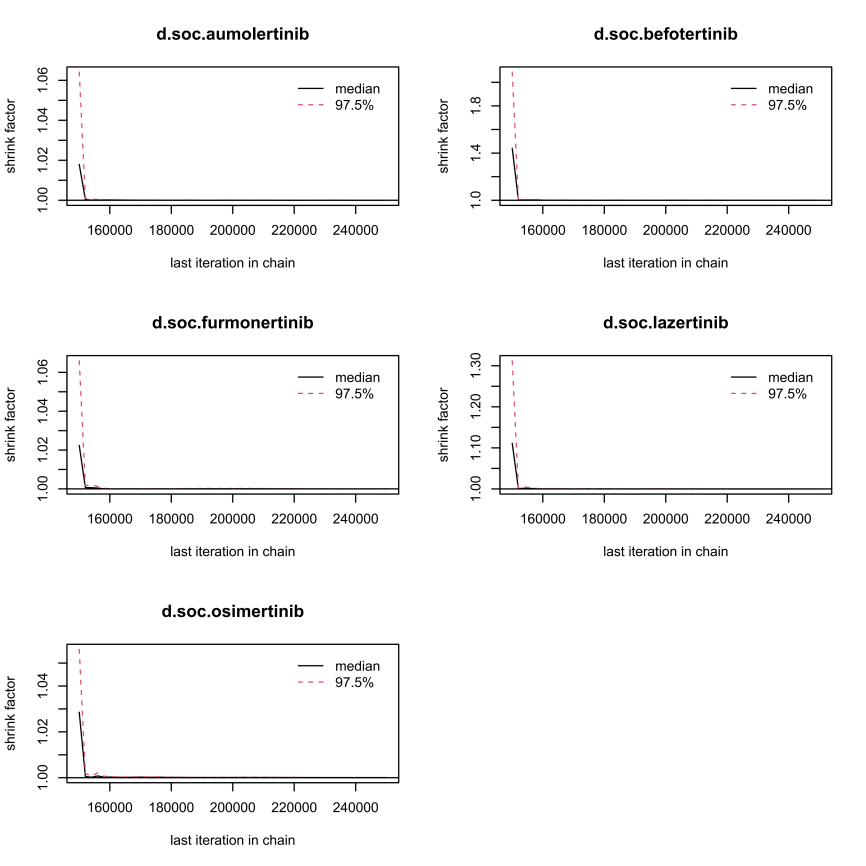

Supplement: Supplementary file 1 — Supplemental Table 1. Comparison of the fit goodness between consistency and inconsistency models based on DIC values in network meta‐analysis. Supplemental Table 2. Node‐splitting analysis of inconsistency. Supplemental Table 3. PRISMA NMA Checklist of Items to Include When Reporting A Systematic Review Involving a Network Meta‐analysis. Supplemental Table 4. Literature Search Strategy (october 23rd, 2024). Supplemental Figure 1. Risk of bias assessments for the included randomized controlled studies according to the Cochrane Risk of Bias Tool 2.0. Supplemental Figure 2. Bayesian ranking profiles of comparable treatments on efficacy and safety for patients with advanced EGFR mutated NSCLC. Supplemental Figure 3. Pooled relative PFS of treatment strategies stratified by EGFR mutation in subgroup network meta‐analysis. Supplemental Figure 4. Pooled relative PFS of treatment strategies stratified by ECOG PS in subgroup network meta‐analysis. Supplemental Figure 5. Pooled relative PFS of treatment strategies stratified by sex in subgroup network meta‐analysis. Supplemental Figure 6. Pooled relative PFS of treatment strategies stratified by smoking history in subgroup network meta‐analysis. Supplemental Figure 7. Pooled relative PFS of treatment strategies stratified by brain metastases in subgroup network meta‐analysis. Supplemental Figure 8. Forest plot of the pooled estimates: Pairwise vs. Network Meta analyses. Supplemental Figure 9. Forest plot of the pooled estimates: Pairwise vs. Network Meta analyses. Supplemental Figure 10. Forest plot of the pooled estimates: Pairwise vs. Network Meta analyses. Supplemental Figure 11. Convergence of the three chains established by inspection of the history feature and the Brooks‐Gelman‐Rubin diagnostic for objective response rate. Supplemental Figure 12. Convergence of the three chains established by inspection of the history feature and the Brooks‐Gelman‐Rubin diagnostic for overall survival. Supplemental Figure 13. Convergen [file MCO2-6-e70393-s001.docx]
